# Unraveling Charge-Transfer States and Their Ultrafast Dynamics in Artificial Light-Harvesting Complexes

**DOI:** 10.1021/acsphyschemau.5c00098

**Published:** 2026-01-12

**Authors:** Luís Gustavo Teixeira Alves Duarte, Iker Lamas, Dominik Bäuerle, Saeed Shareef, Renato D. Cunha, Carles Curutchet, Mariano Curti, Elisabet Romero

**Affiliations:** 1 Institute of Chemical Research of Catalonia (ICIQ-CERCA), Barcelona Institute of Science and Technology (BIST), Avda. Països Catalans 16, Tarragona 43007, Spain; 2 Departament de Química Física i Inorgànica, Universitat Rovira i Virgili, C/Marcel·lí Domingo s/n, Tarragona 43007, Spain; 3 Departament de Farmàcia i Tecnologia Farmacèutica, i Fisicoquímica, Facultat de Farmàcia i Ciències de l’Alimentació, Universitat de Barcelona (UB), Av. Joan XXIII 27-31, Barcelona 08028, Spain; 4 Institut de Química Teòrica i Computacional (IQTC-UB), Universitat de Barcelona (UB), Barcelona 08007, Spain; 5 Aix Marseille Univ, CNRS, ICR, Marseille 13397, France

**Keywords:** de novo protein design, artificial light-harvesting complexes, excitonic coupling, Stark spectroscopy, transient absorption spectroscopy

## Abstract

Photosynthesis relies on highly organized pigment–protein complexes in order to store sunlight energy as biochemical energy. These complexes capture light with remarkable efficiency and are responsible for ultrafast charge separation within a finely tuned energy landscape provided by the protein environments, producing one of nature’s most sophisticated energy conversion systems. Inspired by nature, *de novo* designed proteins have been proven to be versatile platforms to emulate the function of natural light-harvesting complexes and reaction centers. With Stark and ultrafast transient absorption spectroscopies, we explored the exciton and charge-transfer (CT) mixing, as well as the excited-state dynamics, of a chlorophyll *a* analogue (Zn-pheophorbide *a*) in dimers formed within 4-α-helix bundles whose design was previously guided by molecular dynamics simulations. Due to dimerization, we observe an increase in the CT character of the excitonically coupled dimers’ excited state in comparison to monomeric ZnP. Furthermore, additional nonradiative relaxation pathways, together with the formation of transient species absent in monomeric systems, were observed for the dimers. We demonstrate that *de novo* designed proteins can replicate key features of photosynthetic energy conversion, serving as tunable scaffolds for optimizing light-harvesting processes. Ultimately, these systems have promising applications including photovoltaic cells and biomedical treatments based on sustainable materials.

## Graphical abstract

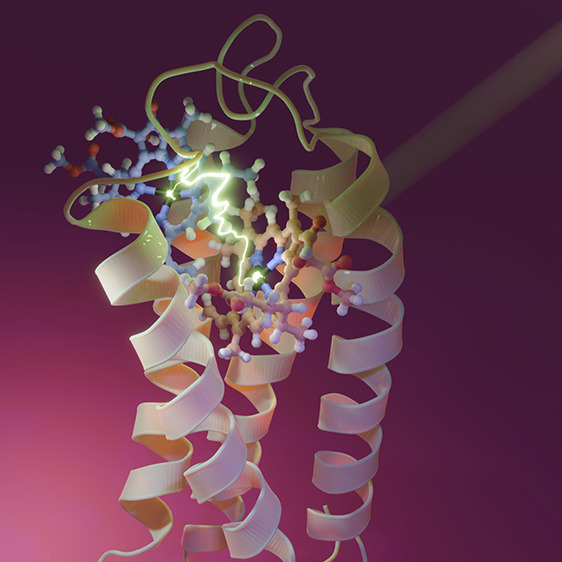

## Introduction

In nature, photosynthesis is an extremely effective process in capturing and converting sunlight energy, with light reactions that reach near-unity quantum efficiency.^1–4^ Photosynthetic organisms make use of sunlight via densely packed arrays of pigments embedded within transmembrane proteins in the so-called light-harvesting complexes (LHCs) and reaction centers (RCs).^5–7^ These complexes and supercomplexes formed by both LHCs and RCs have large absorption cross sections in the visible and near-infrared regions of the electromagnetic spectrum by containing tens to hundreds of pigments, mostly (bacterio)­chlorophylls, carotenoids, or phycobilins, which undergo processes as light absorption and energy transfer to maximize the utilization of sunlight energy by funneling it from the LHCs to the RCs, where the charge separation process is initiated in special molecular dimers.^8^ These dimers, constituted by excitonically coupled (bacterio)­chlorophyll molecules which possess excited states with CT character,^9–11^ trigger the sunlight conversion into electrochemical energy. Remarkably, photosynthetic proteins offer the means to accommodate a large density of pigments while simultaneously providing an energy landscape that increases pigment photostability, favors the energy transfer between pigments and tunes excitation quenching caused by aggregation. Additionally, the protein structure assists in the arrangement of molecules in a cascade of redox potentials that leads to stable charge separation.^12^

The relevant roles exerted by photosynthetic proteins motivate the quest for their replication for applications in sustainable energy production by photonic devices^13^ and biomedicine with photodynamic therapy protocols,^14^ among others.^15^ In this regard, the de novo design of synthetic proteins has proven to be a promising approach to emulate natural photosynthesis with the possibility of composing simpler versions of natural proteins while maintaining tunability of their essential structure–function relationships.^16^ With this approach, water-soluble synthetic proteins can be produced and easily mutated to accommodate different cofactors to create artificial LHCs and RCs. For this work, we employed synthetic proteins that are originally constituted by a structural motif of nonpolar/polar amino acid heptad repeats that fold as α-helical bundles with the nonpolar and polar residues facing the bundle’s core and exterior, respectively.^17,18^ The protein’s hydrophobic core makes them a suitable host of tetrapyrrole based cofactors, providing a platform for supramolecular assemblies for light-harvesting applications.^19^ Bundles with 4-α-helixes, both as single-chain monomers^20^ and tetramers of single-helix proteins,^21,22^ are the most reported design for emulating the interior of many redox proteins,^23^ enzymes and photosynthetic reaction center complexes.^16,24,25^

The choice of the protein’s amino acid sequences allows the arrangement of the cofactors in different positions and proportions with the intention of impacting their final electronic structures.^17,26^ Previous studies demonstrated that de novo designed protein helices can improve cofactor redox activity by accelerating the electron transfer kinetics.^21^ Besides, Hobbs et al. demonstrated that synthetic proteins containing tetrapyrrole derivatives can act as sensitizers in dye-sensitized solar cells, offering better performance in comparison to the devices made only with tetrapyrroles, on account of a microenvironment that stabilizes charge-separated states for three times longer.^13^

One strategy to achieve such behavior concerns the incorporation of cofactors in close proximity to each other to induce excitonic interactions.^16,24^ As observed in natural photosynthetic proteins, such coupling leads to the redistribution of the energetically accessible landscape of states, changing the photophysical and chemical behavior in the electronic excited state. Here, we investigate the CT character and the ultrafast excited state dynamics of our previous de novo designed proteins with histidine residues ligating the chlorophyll *a* (Chl *a*) analogue Zn-pheophorbide *a* (ZnP, for structure see Figure 1a) as cofactor in two pairs of excitonically coupled dimers within a monomeric 4-α-helix-bundle. Our protein scaffolds are built on the basis of the de novo protein BT6, which contains four histidine binding sites in adjacent helices, two at the top and two at the bottom of the protein structure, initially designed as a flexible platform for emulating natural redox processes binding two hemes as cofactors.^26^ The BT6–2H→2A mutant (Figure 1b) was designed to bind two noninteracting ZnPs to serve as control sample,^27^ with two ZnP-binding histidines at opposed ends of BT6 replaced by alanines. On the other hand, the BT6–4E→4K mutant (Figure 1c) was developed to enforce the binding of four ZnP chromophores (which did not occur in the original BT6 design), leading to two excitonically coupled dimers.^27^ In BT6–4E→4K, four glutamate residues, each of them contiguous to a histidine, are replaced with a positively charged lysine residue, with the goal of enhancing electrostatic interactions with the polar side of the chromophore (and thus increasing its binding energy). An additional BT6 mutant, termed 4L→4A (Figure 1d), was designed with the intention of improving the binding energy of ZnP to the protein, in this case by reducing concurrent steric hindrances with the replacement of four leucine residues by four alanines. By means of Stark and ultrafast transient absorption spectroscopies, we characterize the CT character of the ZnP excited states and their ultrafast excited state dynamics, respectively, for the monomeric ZnP in BT6–2H→2A and for both the excitonically coupled dimers in BT6–4E→4K and BT6–4L→4A. Our results demonstrate the occurrence of CT states mixing with excitonic states for the ZnP dimers within BT6–4E→4K and BT6–4L→4A, a finding that is corroborated by transient absorption experiments, where faster nonradiative deactivation mechanisms than in the monomeric case and short-lived radical formation are observed.

**Figure 1:**
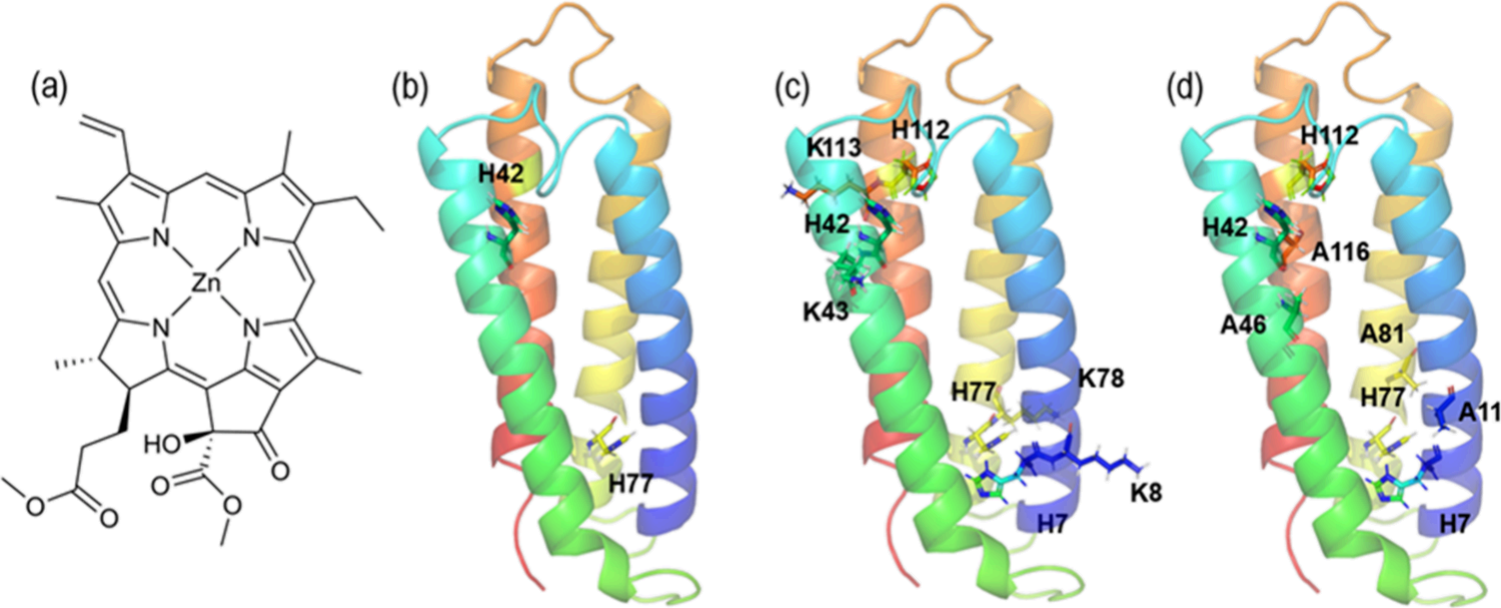
(a) Molecular structure of ZnP. (b) 2H→2A mutant, where one histidine at each side of BT6 has been replaced by an alanine. Highlighted are the two remaining histidines in positions 42 and 77. (c) 4E→4K mutant, where each glutamate contiguous to a histidine in BT6 has been replaced by a lysine. Highlighted are the four histidines in positions 7, 42, 77, and 112, and lysines in positions 8, 43, 78, and 113. (d) 4L→4A mutant, where each leucine contiguous to a histidine in BT6 has been replaced by an alanine. Highlighted are the four histidines in positions 7, 42, 77, and 112, and alanines in positions 11, 46, 81, 116.

## Results and Discussion

### Stark Spectroscopy

Stark spectroscopy, also known as electrochromism or electroabsorption, monitors the changes on the absorption spectrum induced by an externally applied electric field, and thus, it is an extremely sensitive technique to the electronic distribution changes of chromophores upon excitation. The Stark spectrum, defined as the difference between the absorption in the presence and absence of the externally applied electric field (*Abs*_*F*_on__ – *Abs*_*F*_off__), provides detailed information about two crucial molecular parameters: the change in polarizability (Δα) and the change in dipole moment strength (Δμ) between the ground and excited states associated with an electronic transition. Here, we apply Stark spectroscopy to investigate the CT character of the chromophores’ excited states which manifests as large Δμ values of the excitonic transitions.^28,29^

The Stark spectra for ZnP complexes of the 2H→2A, 4E→4K and 4L→4A proteins in the Q bands are shown in Figure 2 (for the full spectra of all samples, see Figures S1–S3). For all complexes, the strongest Stark signals are observed in the Q*_*y*_* region (≈ 650 – 700 nm). We focus our attention on this range since it contains the lowest excited states that, as in photosynthetic complexes, are involved in energy and electron transfer processes.

**Figure 2:**
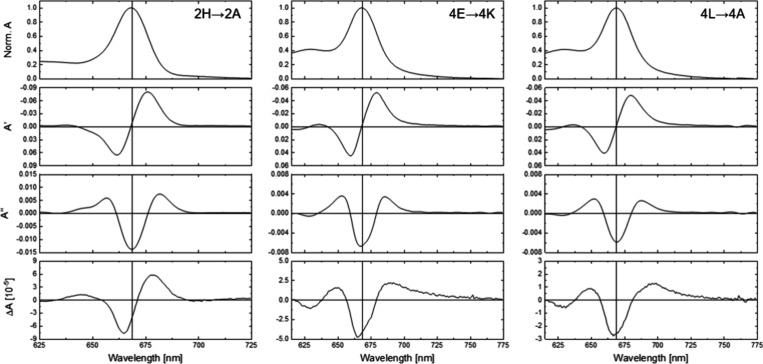
Normalized Absorption (Norm *A*) spectra at 77 K, its first (*A*’) and second derivatives (*A*”), and Stark spectrum (**Δ** *A*) for 2H→2A (left), 4E→4K (center) and 4L→4A (right).

As stated above, the binding of two ZnP molecules to the 2H→2A mutant leads to two noninteracting chromophores, as evinced from previous circular dichroism spectra and computational results.^27^ Therefore, 2H→2A serves as a benchmark to determine the Stark response for ZnP monomers. The comparison of the Stark spectrum measured for 2H→2A with the first and second derivatives of its absorption spectrum shows greater similarity to the first derivative. This indicates that the change in polarizability upon excitation dominates the Stark signal, as opposed to the contribution from the change in dipole moment, which is characterized by a second derivative line shape.^30^ The point of zero-crossing, however, is slightly red-shifted in the Stark spectrum (672 nm versus the 669 nm in the first derivative), indicating some contribution to the Stark spectrum from the second derivative as well.

The results for the 4E→4K and 4L→4A mutants (Figure 2), where four chromophore molecules are bound as two excitonically coupled dimers, are markedly different. On the one hand, the Q*_*y*_* absorption is significantly broader than for 2H→2A, which we attribute to excitonic coupling in 4E→4K and 4L→4A causing a splitting of the main monomer band (see also the fits below on Figure 3).^27^ Additionally, the Q*_*y*_* Stark spectrum now resembles the second derivative of the absorption, signifying a stronger contribution from the change in dipole moment between the ground and excited states. Like in the monomer case, this contribution is not unique: the minimum in the Stark spectrum is at 665 nm, while that of the second derivative is at 669 nm. A significant contribution from the first derivative likely causes this blueshift in the Stark spectrum.

**Figure 3:**
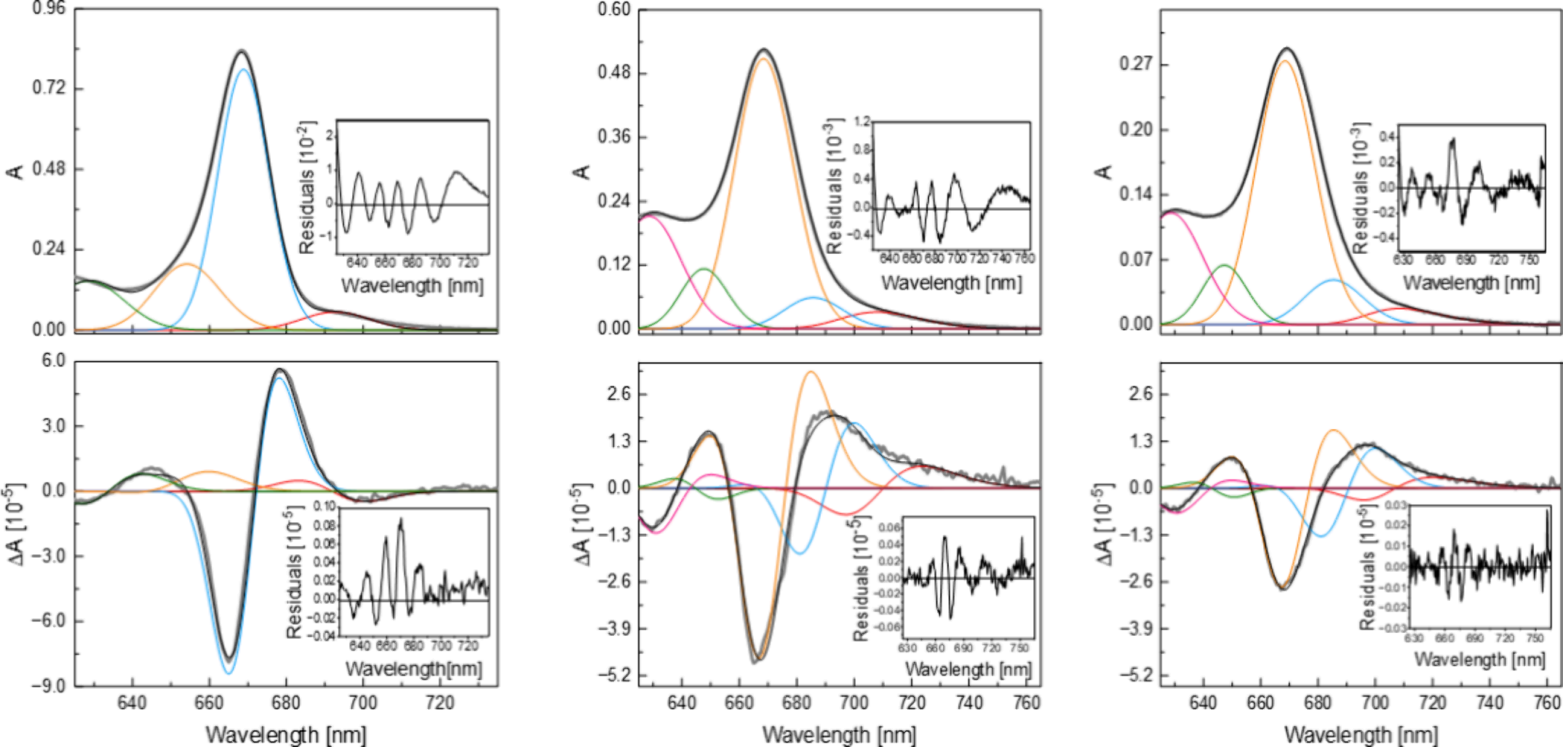
Spectral fittings for 2H→2A (left), 4E→4K (center), and 4L→4A (right). Top: absorption spectra – experimental: gray dots; fit: black line; fit components: red, blue, yellow, green and pink lines. Bottom: Stark spectra – experimental: gray line; fit: black line; fit components: red, blue, yellow, green and pink lines. Fit residuals are shown as insets.

A close inspection of the absorption spectra of 2H→2A, 4E→4K and 4L→4A at 77 K reveals a feature beyond the absorption maxima in the 700–750 nm red region that is less prominent at room temperature.^27^ This feature is ascribed to a small fraction of an identified chromophore impurity (≈10%), and thus it will not be discussed further (see supplementary note 1, Figures S4 to S10).

To obtain quantitative information from the Stark spectra of the complexes, we performed fittings based on Liptay’s formalism (Figure 3).^30,31^ In this scenario, each electronic transition in the absorption spectra is initially fitted as a skewed Gaussian band, parametrized by its amplitude *A*_0_, center ν_0_, width σ, and skewness γ, as follows

$$A\left(\nu ;A_{0},\nu_{0},\sigma ,\gamma \right)=\frac{A_{0}}{\sqrt{2\pi }\sigma }\exp\left[\frac{-{\left(\nu -\nu_{0}\right)}^{2}}{2\sigma^{2}}\right]\left\{1+\mathrm{erf}\left[\frac{\gamma \left(\nu -\nu_{0}\right)}{\sqrt{2}\sigma }\right]\right\}$$ 1

where *erf* is the error function over the real numbers. The Stark signal at a particular frequency Δ*A*(ν) is then fitted as the sum of the zero, first and second order derivatives of the respective absorption spectrum as

$$\mathrm{\Delta A}\left(\nu \right)={\left(fF\right)}^{2}\left\{A_{\chi }A\left(\nu \right)+B_{\chi }\nu \frac{d}{d\nu }\left(\frac{A\left(\nu \right)}{\nu }\right)+C_{\chi }\nu \frac{d^{2}}{d\nu^{2}}\left(\frac{A\left(\nu \right)}{\nu }\right)\right\}$$ 2

where *F* is the external electric field, *f* is the internal field correction factor (a correction for the magnitude of the unknown local field present at the chromophore position), *h* is the Planck constant, and *c* is the speed of light. *A*_χ_ is the transition polarizability, which is related to the contribution of the external electric field to the chromophore transition dipole moment. *B*_χ_ and *C*_χ_ are related to the differences of the molecular polarizability tensor and the electric dipole moment between ground and excited states, respectively. When the angle between the electric field vector of the measuring light and the direction of the applied electric field on the sample cell is equal to the magic angle, *A*_χ_ becomes negligible, and *B*_χ_ and *C*_χ_ can be expressed as

$$B_{54.7{}^{\circ}}=\frac{Tr\left(\Delta \alpha \right)}{6hc}$$ 3

$$C_{54.7{}^{\circ}}=\frac{{\Delta \mu }^{2}}{6h^{2}c^{2}}$$ 4

We employed four components to fit the absorption of the 2H→2A complex in the 625 – 770 nm region, representing the main Q*_*y*_* absorption band (669 nm), two vibronic bands (635 and 653 nm), and a low-amplitude band corresponding to the red-absorbing impurity (697 nm). The absorption fitting of 4E→4K and 4L→4A includes an additional component, to account for the excitonic splitting of the Q*_*y*_* state upon dimer formation, leading to excitonic components at 685 and 667 nm, which we are going to address as low and high excitonic states.

Using these sets of components, both absorption and Stark spectra are simultaneously fitted to a good tolerance, as demonstrated by the respective residual plots (Figure 3). Inspection of the different contributions confirms the above qualitative analysis for the Q*_*y*_* bands: the Stark spectrum for 2H→2A displays a larger first-derivative contribution, while that for 4E→4K and 4L→4A are characterized by stronger second-derivative contributions.

The parameters obtained from these fittings are collected in Table 1. The Q*_*y*_* transition of the 2H→2A complex is characterized by a change in polarizability of *Tr­(*Δα*)*_*2H→2A*_ = 10.5 Å^3^ *f* ^–2^, while its corresponding change in dipole moment is Δμ_*2H→2A*_ = 0.64 D*f* ^–1^. For the 4E→4K and 4L→4A complexes, the high exciton component at 667 nm displays values of *Tr­(*Δα*)*_*4E→4K*_ = 15.0 Å^3^ *f* ^–2^and Δμ_*4E→4K*_ = 1.17 D*f* ^–1^, and *Tr­(*Δα*)*_*4L→4A*_ = 12.0 Å^3^ *f* ^–2^and Δμ_*4L→4A*_ = 1.18 D*f* ^–1^; whereas the low exciton component at 685 nm displays values of *Tr­(*Δα*)*_*4E→4K*_ = 117.7 Å^3^ *f* ^–2^and Δμ_*4E→4K*_ = 1.79 D*f* ^–1^, *Tr­(*Δα*)*_*4L→4A*_ = 94.1 Å^3^ *f* ^–2^ and Δμ_*4L→4A*_ = 1.66 D*f* ^–1^. The significantly higher changes in dipole moment for the excitonically coupled dimers with respect to the monomeric ZnP in the 2H→2A complex (two and three times for the high and low exciton component, respectively), and the occurrence of extra electronic transitions in the spectra of 4E→4K and 4L→4A complexes resulting in a more entangled Stark spectra with second-derivate shapes, are strong evidence for the mixing of excitonic and CT states upon ZnP dimerization.

**Table 1:** Parameters Obtained from the Simultaneous Fitting of Absorption and Stark Spectra Employing Liptay’s Formalism^a^

Sample	Center [nm]	Area	Width	*Tr (*Δα*)* [Å^3^ *f* ^–2^]	Δμ [D*f* ^–1^]
2H→2A	697	25.0	200.0	–57.5	0.55
	**669**	**291.9**	**149.9**	**10.5**	**0.64**
	653	98.4	200.0	14.3	0.26
	635	88.4	300.0	21.8	1.22
4E→4K	708	22.0	363.2	126.9	1.04
	**685**	**32.0**	**217.5**	**117.7**	**1.79**
	**667**	**291.2**	**231.2**	**15.0**	**1.17**
	650	54.9	199.9	–9.4	0.37
	636	151.9	350.0	–9.8	1.1
4L→4A	708	11.2	382.1	105.5	0.99
	**685**	**25.7**	**218.5**	**94.1**	**1.66**
	**667**	**158.4**	**231.2**	**12.0**	**1.18**
	650	28.8	193.7	–9.7	0.46
	636	84.0	350.0	–8.0	1.09

a Absorption bands of interest are highlighted in bold.

For comparison with our results, the corresponding values for Chl *a* in monomeric form are *Tr­(*Δα*)*_*Chla*_ = 2.2 Å^3^ *f* ^–2^and Δμ_*Chla*_ = 0.95 D*f* ^–1^, while for Chl *a* dimers these increase to *Tr­(*Δα*)*_*Chla‑dimer*_ = 93 Å^3^ *f* ^–2^and Δμ_*Chla‑dimer*_ = 5.2 D*f* ^–1^(≈ 40-fold for *Tr­(*Δα*)* and ≈ 5-fold for Δμ).^28,32^ Chl *a* dimer formation thus leads to a larger increase in dipole moment change than the one observed here, indicative of a strong CT character of the Chl *a* dimer lowest exciton component excited state. Comparatively, we observe a more modest increase, from 0.64 *Df* ^–1^ in 2H→2A to 1.17 D*f* ^–1^/1.18 D*f* ^–1^ (high exciton) and 1.79 D*f* ^–1^/1.66 D*f* ^–1^(low exciton) in 4E→4K and 4L→4A, respectively. Nevertheless, as discussed for the time-resolved data below, this relatively small change in the electronic properties of the dimers is enough to yield distinct excited state dynamics.

Our observations can be correlated to the changes in dipole moment found previously for 13^2^-OH-Zinc-bacteriochlorophyllide-*a* (ZnBChlide) also bound to synthetic proteins, and used as a mimetic system of bacteriochlorophyll *a*.^31^ Unlike in ZnP, dimerization of ZnBChlide does not result in CT states with dipole moment changes higher than ZnBChlide monomers when inserted into α-helical bundles of closely related structure to ours (Δμ ≈ 4.0 D*f* ^–1^ for both monomers and dimers). For ZnBChlide, most likely the keto group present in the same position of the vinyl group of ZnP molecular structure is already responsible for inducing higher dipole moments and dipole moment changes. The same trend was observed for BChl *a* monomers when compared to Chl *a* monomers (Δμ_*BChla*_ ≈ 2.0 D*f* ^–1^ and Δμ_*Chla*_ = 0.95 D*f* ^–1^).^28,32^ Hence, dimerization of ZnP results in a greater relative change in Stark signal than for ZnBChlide in the artificial bundles. Therefore, ZnP dimer is an interesting system for exploring how de novo designed proteins can enhance the CT character of the cofactors’ electronic states, either by means of altering local electric fields or by shaping the protein structures to induce specific dimer conformations.

### Transient Absorption Spectroscopy

Aiming to gain insights on the excited state dynamics of the designed assemblies and to further confirm the observations made in the Stark spectroscopy experiments, the relaxation routes available in these chromophore–protein complexes were studied by means of broadband transient absorption spectroscopy (BB-TAS). Figure 4a shows the steady-state absorption and emission spectra of the ZnP chromophore and the different assemblies, together with the laser spectrum employed as pump and probe pulses in the BB-TAS measurements. As it can be observed in Figure 4, the time-resolved experiments were conducted to explore the photodynamical behavior of the samples following photoexcitation in the Q*_*y*_* absorbance band (S_1_), which is assumed to be the most relevant one for tracking processes in the readily accessible ps-ns time scales of the experiment, due to the fact that excitation in the Soret (S_2_) region typically leads to ultrafast internal conversion (IC) to the Q-bands on subps time scales.^33–43^

**Figure 4:**
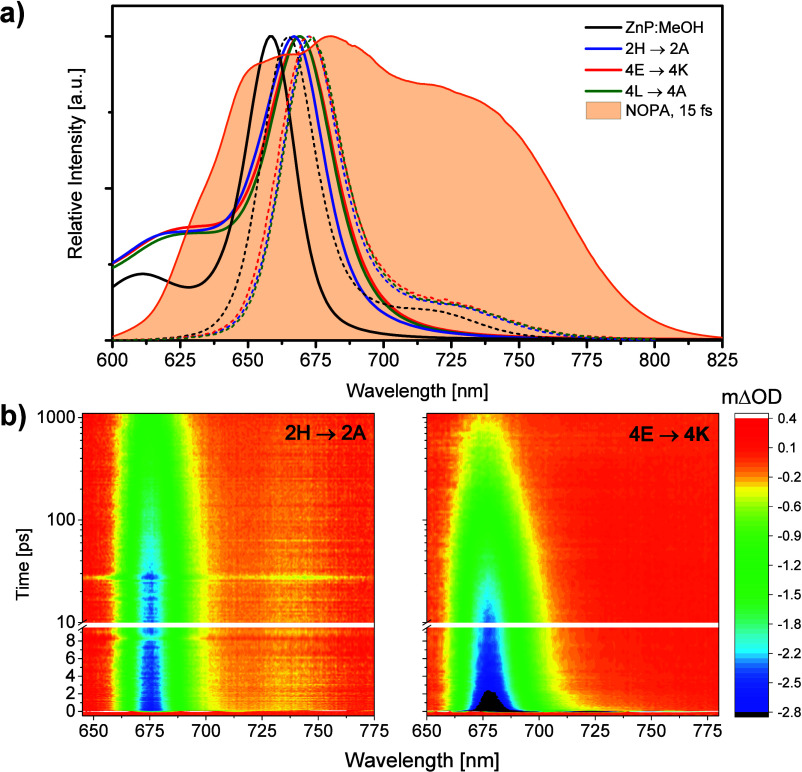
(a) Absorption (solid lines) and emission (dashed lines) spectra of ZnP in methanol (black lines), and 2H→2A (blue lines), 4E→4K (red lines) and 4L→4A (green lines) chromophore–protein complexes, together with the spectrum of the noncollinear optical parametric amplifier (NOPA) output employed in the BB-TAS measurements. (b) BB-TAS data at magic angle of 2H→2A and 4E→4K.

The BB-TAS full data sets collected in the magic angle configuration between pump and probe for 2H→2A and 4E→4K complexes are displayed in Figure 4b. Due to the analogous photodynamical behavior of both dimer-containing assemblies 4E→4K and 4L→4A, as also observed in steady-state spectroscopy, the BB-TAS results recorded for 4L→4A are shown in Figure S11, and hereafter we will focus on the comparison between 2H→2A and 4E→4K complexes. The BB-TAS spectra at selected time-delays for 2H→2A and 4E→4K displayed in Figure 5a show the following general trends. At early times the BB-TAS spectra of both assemblies exhibit a prominent negative feature with the main peak around 675 nm, which corresponds to the overlapping contributions of ground state bleach (GSB) and Stokes-shifted stimulated emission (SE) of the initially populated Q*_*y*_* that extends to longer probe wavelengths, covering the 715–760 nm range with SE into vibrationally hot ground states, which is also visible in the recorded steady-state fluorescence spectra (see dashed lines in Figure 4a). The main band recovery is significantly faster for the assemblies that contain excitonically coupled dimers, while in 2H→2A hardly any recovery occurs during the first tens of ps. Additionally, there is a significant reduction in SE signal during the first tens of ps for the chromophore dimer-containing assemblies, while 2H→2A maintains a similar ratio between the red SE and the main band throughout the observation window (1 ns). Interestingly, the formation in tens of ps of a small positive contribution that extends from 712 nm up to the detection limit at 780 nm is perceptible only for the dimer-containing assemblies. This broad photoinduced absorption (PIA) on the red spectral region decays in hundreds of ps. The time-traces recorded at two characteristic wavelengths are shown in Figure 6a: 675 nm shows the main band dynamics and 735 nm corresponds to SE present in both systems and the PIA only observed in dimer-containing assemblies. Consequently, unlike in 2H→2A, access to an additional nonradiative relaxation pathway and the formation of new species in the excited state seem to be available for the complexes with excitonically coupled dimers, which will be discussed in detail below.

**Figure 5:**
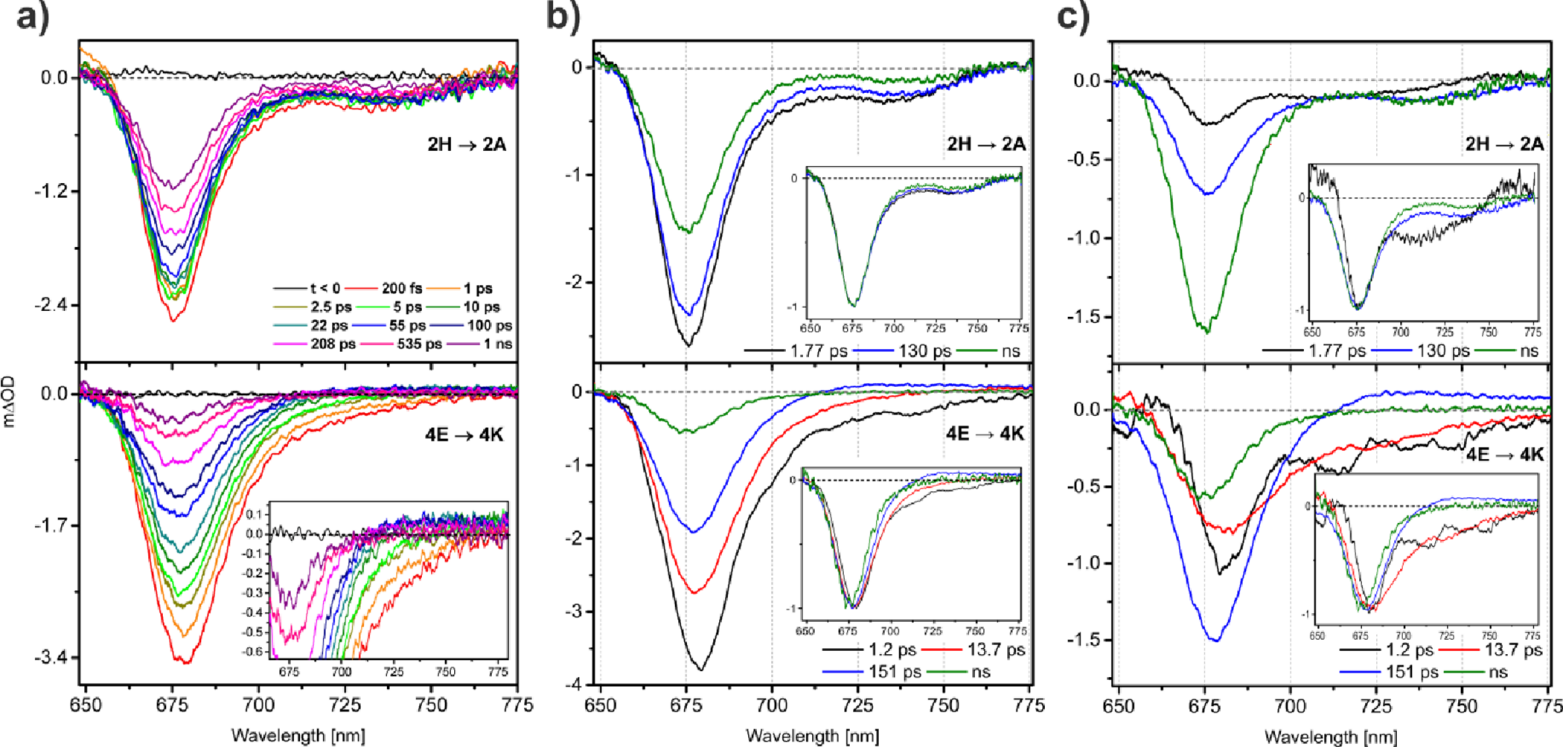
(a) BB-TA spectra recorded at the magic angle configuration at selected time-delays for 2H→2A (upper panel) and the 4E→4K (bottom panel) complexes with an inset showing a zoom-in into the SE region. (b) Evolution-associated difference spectra (EADS) and (c) decay-associated difference spectra (DADS) for the 2H→2A (upper panel) and 4E→4K (bottom panel) complexes, insets show normalized spectra.

**Figure 6:**
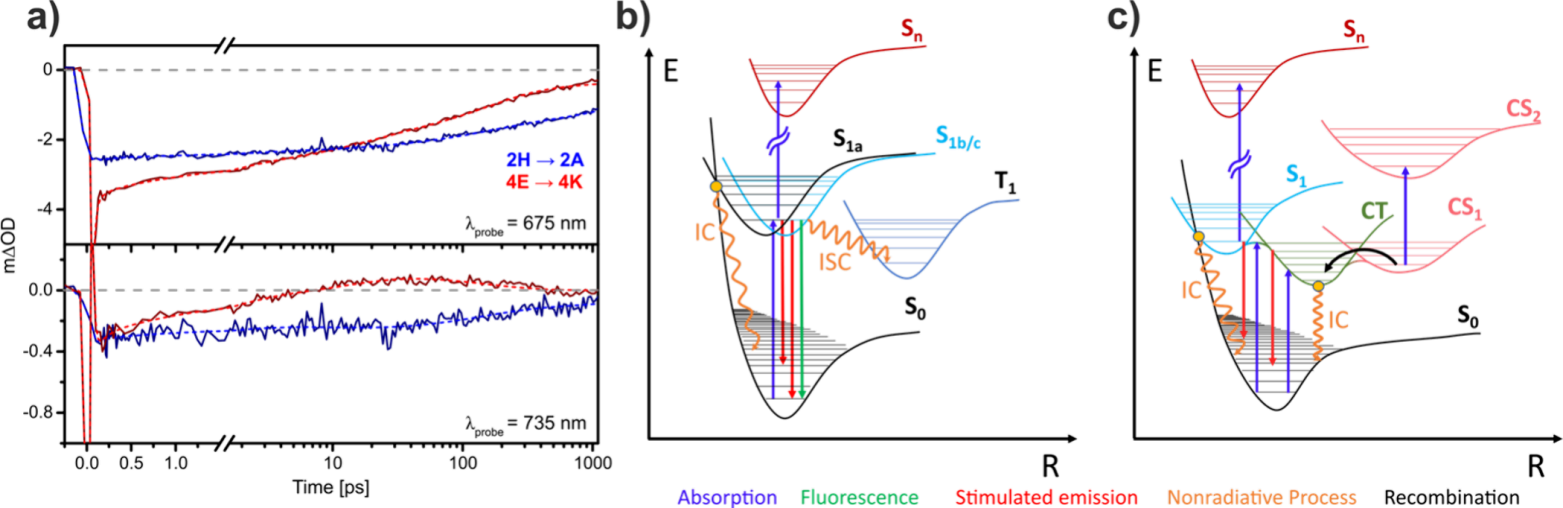
(a) Comparison of recorded time-traces for 2H**→**2A (blue solid lines) and 4E**→**4K complexes (red solid lines) at 675 (top panel) and 735 nm (bottom panel) probe wavelengths. The dashed lines correspond to the global analysis fits. (b) Energy level scheme and the expected transitions in the monomer-containing 2H**→**2A assembly: Following photoexcitation in the Q_*y*_ (S_1_), the system undergoes nonradiative intersystem crossing to the triplet manifold, and internal conversion or fluorescence to the ground state. The specific configurations ***a*** (1.77 ps), ***b*** (130 ps) and ***c*** (3.7 ns) of molecules in the sample determine which decay channel is dominant, see main text. (c) Energy level scheme and the expected transitions for the dimer-containing assemblies: After excitation of the excitonic S_1_ state, either direct deactivation via internal conversion occurs, or the mixing with a charge-transfer (CT) state favors movement along the nuclear coordinate, both in about 1.2 ps. Subsequently, nonradiative relaxation to the ground state or conversion into a metastable charge-separated (CS) state in 14 ps, with photoinduced absorption that recombines in about 151 ps are possible. A small fraction in the sample resembles the monomeric case shown in (b) and decays in 3.7 ns. In (c), intersystem crossing to the triplet manifold is not shown for clarity but is expected to occur at various points of the PES.

Aiming to identify spectral features that can be assigned to specific processes along the deactivation routes of these species, a global analysis was performed, in which the BB-TAS data was fitted using an unbranched unidirectional (sequential) or parallel kinetic model. The BB-TAS spectra at selected time-delays for 2H→2A and 4E→4K complexes are displayed in Figure 5a, while Figure 5b,c depict the extracted time-components and evolution-associated difference spectra (EADS; sequential model) and decay-associated difference spectra (DADS; parallel model), respectively. The EADS provide a compact representation of the spectral evolution (as given by the raw data, as the selection of spectra shown in Figure 5a), while the DADS provide information about the spectral changes on a certain time scale, assuming a parallel decay of independent model compartments. In the DADS, negative amplitudes indicate the recovery of GSB/SE or the rise of PIA features, whereas positive amplitudes reflect the formation of GSB/SE or the decay of PIA signals.^44^ For the BB-TAS data of 2H→2A, three exponential time-components provided a satisfactory fit, while in the case of 4E→4K an additional component was required. It is important to mention that both kinetic models often do not directly map on the physical system and the fitted spectra and concentrations therefore do not have a 1:1 correspondence with species in solution. The impact of this assumption on the interpretation will be discussed in more detail for each case below.

The photodynamics of 2H→2A is characterized by three time-components: τ_*1*_ = 1.77 ps, τ_*2*_ = 130 ps and τ_*3*_ ≫ 1 ns. The latter component cannot be precisely determined due to the limit of our observation window (1 ns). However, the value of this long-lived constant is fixed to 3.7 ns, which is the fluorescence lifetime measured in time-correlated single-photon counting (TCSPC) experiments (Figure S12 and Table S1). As it is visible in both EADS and DADS (upper panels of Figure 5b,c, respectively), all three time-constants contribute to the GSB recovery and disappearance of SE. The number of lifetimes and mostly negative sign of the DADS are an indication that the solution is a heterogeneous mixture of species, since

i. a typical decay of a chromophore is expected to occur with a single effective lifetime that results from the inverse of the sum of the rates for the different decay channels accessible to a relaxed excited state.
ii. spectral changes at constant total population of bright excited states, like rearrangement of the environment and vibrational cooling, as well as energy transfer processes like Förster resonance energy transfer, typically result in significant positive contributions in the DADS that compensate for the loss of GSB and SE in other regions. While we assume that such effects might be present in the samples and contribute to the fitted DADS, we can exclude that their contributions are significant enough to isolate them.

The spectral shape remains almost unaltered throughout the experimental time scale, which is observable by normalizing the EADS, as shown in the insets of Figure 5b (upper panel). In light of the heterogeneity assumption, this allows us to assume that the different supposed species in solution strongly resemble each other spectrally. This means that analysis with a parallel decay model might be a decent representation of the species spectra in this case, as we do not see any direct evidence for sequential evolution of species and several independent compartments have to be employed to explain the multiexponential kinetics.

In the DADS, the first component (τ_*1*_ = 1.77 ps) contributes to about 10% of total signal decay and shares the main GSB minimum at about 676 nm and red edge with the slower components, consistent with an assumption of a similar 0–0 transition energy for all three species. A stronger relative contribution of SE in the vibronic wing reports on a potentially more shifted excited state potential energy surface (PES) of this species, causing increased Frack-Condon overlaps for transitions into vibrationally hot ground states. Similarly, the deviation from the blue edge of the slower components can be explained by a more pronounced contribution of excited state absorption (ESA) into the Soret band. We derive from the observations that an excited state shifted along the nuclear coordinate might give access to fast nonadiabatic dynamics that return the small ensemble of molecules in this configuration to the ground state in a few ps.

The second (τ_*2*_ = 130 ps) and third (τ_*3*_ = 3.7 ns) components seem to represent decays from an almost identical excited state, but along different dominant decay channels. This can be rationalized by comparing the DADS of both lifetimes (blue and green lines in Figure 5c, upper panel), in which the shape and intensity of the SE happen to overlap, while the main bleach intensity is close to half for τ_*2*_. When normalized (see the inset in Figure 5c, upper panel), the DADS agree perfectly in the main bleach region but differ in the relative contribution of the SE band on the red side. All observations are readily explained by identifying the second component as mostly loss of stimulated emission from the transient absorption signal due to intersystem crossing (ISC) of the excited state to the triplet manifold, as reported for Zn porphyrins and ZnBChlides.^33,36,37,45^ Triplets do not significantly contribute to signals in the observation window, while also not leading to recovery of ground state population due to the triplet state lifetimes in the microsecond range.^46–47^ For the τ_*3*_ time-component, additional loss of intensity in the main band can be explained by including fluorescence as a de-excitation pathway, which besides leading to the same loss of SE as in the τ_*2*_ component, additionally repopulates the ground state. It seems therefore that these two species are mainly distinguished by their ISC rate, which is significantly higher for the τ_*2*_ = 130 ps component.

The ratio of ISC and fluorescence rates determines how much signal decay leads to ground state recovery vs triplet formation on the respective time scale. Since the parallel decay model producing DADS assumes equal initial population of both species, and they both contribute similarly to the disappearance of the red SE band, the most extreme interpretation possible is a purely fluorescent decay for the ns τ_*3*_ component and an identical share of the second and third species in solution. A much more realistic assumption, however, is that the share of the partially fluorescent species in solution is the largest, while the ISC rate remains significantly larger than the natural fluorescence rate in these species, meaning loss of SE is the dominant contribution for τ_*3*_ DADS.^45^

An important point is that these results for 2H→2A are qualitatively identical to those obtained for ZnP in methanol solution, where the chromophores exist as monomers.^27^ Thus, although we cannot entirely rule out protein oligomerization, all the available data indicates that interprotein chromophore interactions are negligible, and both ZnP chromophores in 2H→2A effectively behave as isolated. This is in agreement with previous work on the same or related systems, including TA and Stark experiments.^24,26,27,31^

The global analysis of the 4E→4K complex with four components yields the following lifetimes: τ_*1*_ = 1.2 ps, τ_*2*_ = 13.7 ps, τ_*3*_ = 151 ps and τ_*4*_ ≫ 1 ns. Analogously to 2H→2A, the long-lived τ_*4*_ time-component is fixed to 3.7 ns in order to account for the fluorescence lifetime recorded in TCSPC measurements (see Figure S12 and Table S1). As shown in the bottom panel of Figure 5b, the τ_*1*_ to τ_*4*_ evolution displays more significant spectral changes than in the 2H→2A assembly, such as a gradual blueshift of the main band, a SE band reaching closer toward the main bleach (absent in τ_*3*_), and the formation and subsequent decay (from τ_*3*_ to τ_*4*_) of PIA, between 710 nm and the long wavelength detection limit. Just as in the case of 2H→2A, we assume that the sample might contain a heterogeneous mixture of spectrally similar components due to slight differences in protein conformations, local binding pockets and, particularly in the case of the dimer, relative chromophore orientations. That said, we will lay out in the following that for the first three components we need to assume at least a partial sequential decay process, and that the DADS will not represent simple species spectra scaled by relative concentrations.

The τ_*1*_ component has a very similar lifetime to the one assigned to IC in the 2H→2A assembly. The amplitude of the τ_*1*_ DADS (black line in Figure 5c, bottom panel, with normalized DADS in the inset) is larger than for 2H→2A and reaches almost 25% of the total main band signal. While the fwhm of the main band is comparable to the monomer case, the peak is about 5 nm red-shifted to 681 nm, implying a slightly reduced S_0_-S_1_ energy gap. In analogy to the τ_*1*_ component of the 2H→2A assembly, a stronger relative contribution of SE sideband indicates a shifted PES. Assuming that this component shares its ground state with the τ_*2*_ and τ_*3*_ lifetimes, we can equally conclude that more ESA contributes on the blue side. We therefore attribute most of this decay to IC from an excited state shifted along the nuclear coordinate that maintains S_1_-like character, while experiencing an energy shift and minor PES distortions due to excitonic interactions upon dimerization. We cannot exclude, however, that a part of this excitation evolves along the PES away from its S_1_-like position toward a lower energetic position in the region of S_1_–CT mixing, as also discussed for the following component.

The additional fit component required to describe the 4E→4K data with respect to 2H→2A corresponds to a lifetime of about 14 ps (τ_*2*_). This lifetime is responsible for 20% of the main band recovery and its DADS (red line in Figure 5c, bottom panel) has a larger fwhm than the first component, while the position of its minimum is less clearly defined and lies in the 681–684 nm region. It can be understood as a combination of a GSB shared with τ_*1*_ and τ_*3*_, while having stronger SE contributions close to the red edge of the 0–0 transition. Additionally, the broad negative amplitude reaching up to the red detection limit might partially reflect the formation of the PIA feature starting at 710 nm that is observed in the τ_*3*_ = 151 ps lifetime, which is consistent with the appearance of this band in the τ_*3*_ EADS (blue line in Figure 5b, bottom panel). While it is difficult to disentangle these possible contributions, we note that the larger fwhm and acceptable agreement with the fitted 685 nm position of the low exciton component in steady-state measurements are compatible with a flattened PES due to exciton-CT mixing that also leads to stronger steady-state absorption on the red edge of the Q*_*y*_* band. As mentioned for the first component, part of this state might be populated on the 1.2 ps time scale, as a result of evolution along the nuclear coordinate, away from the Franck–Condon region after excitation, into a coupled exciton-CT state energy valley. In addition, direct excitation into this region as seen in the absorption is assumed to contribute. In either case the state seems to get depopulated about 10 times faster than the fast ISC component in the monomer case, which makes a new mechanism for its decay a reasonable assumption. We believe this mechanism could be both an IC to the ground state and further evolution along the nuclear coordinate into charge-separation.

We assign the τ_*3*_ component to a charge-separated state since the DADS strongly resemble the typical radical pair spectra fitted for the well-studied Chl *a* molecules in reaction centers of natural pigment–protein complexes like photosystem I and II, with the amplitude of the PIA on the red side of the main band reaching about 5% of total bleach intensity.^48–51^ While to the best of our knowledge published absorption spectra of ZnP radicals are not available, we operate on the assumption that the strong resemblance with the cation of Chl *a*, in conjunction with the swift disappearance of the signal, provides the best explanation for our observations. The main alternative for a PIA in this region is triplet absorption, but since such a broad absorption band was not observed in any of the components of 2H→2A and given the published triplet lifetimes,^46^ an unlikely fast triplet quenching mechanism would be necessary to explain the decay in the 151 ps time scale. Charge-recombination into a hot ground state via nonradiative decay is an explanation that we deem more likely. Such a process would be reminiscent of theories about excitation quenching via metastable radical pairs in closed reaction centers of photosystem I, for example.^52,53^

The final τ_*4*_ component (green DADS in Figure 5c, bottom panel) corresponding to the fixed 3.7 ns lifetime, has the shape of a pure GSB signal which is responsible for 10–15% of the total signal amplitude of the main band. The most consistent explanation of this feature within our heterogeneous model is the fluorescence of a small subpopulation in the sample that does not undergo the aforementioned charge-separation and recombination dynamics, since its GSB position at 676 nm is also blue-shifted with respect to the remaining components, and instead coincides with the 2H→2A case. While the lower amplitude is consistent with the reduction in emission quantum yield upon dimerization,^27^ this would still require a small SE vibronic wing as seen in the final component of the monomer fit. Given the lack of an alternative explanation and a small amplitude of this component, the expected vibronic wing feature might be within the noise level of the measurement, and we therefore have to assume that the fitting procedure did not separate the vibronic wing feature from the fluctuating baseline.

As alluded to in the discussion above, it should be noted that no hint of any strong spectral signature ascribable to triplet states is observed in the recorded BB-TAS spectra, but it cannot be ruled out that such a feature lies at longer wavelengths than our detection limit and we implicitly assume that triplet formation occurs due to known observed lifetimes and fluorescence quantum yields of this class of molecules in the lower percent range.^46,54–57^ Similar systems, such as porphyrins, typically exhibit triplet state lifetimes on the microsecond time scale,^45,46,47,55^ so their existence is compatible with the absence of a prezero signal in our BB-TAS experiments at 1 kHz repetition rate.

Results comparable to the magic angle data were obtained with parallel relative polarization between the pump and probe pulses (see supplementary note 2, and Figures S13 and S14). In this case, the loss in signal due to anisotropy effects is not suppressed and therefore a fast decay in the ps time scale reflects the rotational diffusion and/or the reorientation of dipole moments due to state evolution of the investigated systems.^58–60^ Thus, these experiments exhibit an exaggerated strength of GSB recovery and different relative amplitudes of the fitted time-components. Nevertheless, since a similar contribution from the rotational diffusion is expected for all the studied assemblies, it is possible to establish a qualitative comparison of their photophysical behavior.

Schematic representations of the proposed different relaxation mechanisms for 2H→2A and excitonically coupled dimer-containing assemblies are illustrated in Figure 6b,c, respectively. We can conclude that, while we mostly observe decay from similar excited state configurations via different mechanisms in the monomer case, in the case of dimer-containing assemblies the population of the initially photoexcited Q*_*y*_* evolves in a few ps into a mixed exciton-CT state observed in the Stark spectra, giving rise to both faster nonradiative relaxation to the ground state and potentially photoinduced metastable charge-separation.

### Computational Modeling

To obtain atomic-level information on the mutated complexes, we performed molecular dynamic (MD) simulations, spanning four 1-μs replicas for each protein in the holo state (bound to four ZnP chromophores). Representative structures for each system, obtained from a clustering analysis, are displayed in Figure 7. In particular, Figure 7a highlights the main residues stabilizing the chromophore bound to H112 in BT6. This chromophore was simply chosen as an example, as the environment around the others is similar, due to the protein sequence symmetry. The main interactions are van der Waals in origin, and involve residues L116, I38, and W109. Figure 7b,c highlight the mutations in 4E→4K and 4L→4A: in the vicinity of this chromophore, E113 is swapped by K and L116 by A, respectively. An important point is that the mutations do not disturb the interactions with the aforementioned residues, apart from L116 (which is, due to the mutation, not present in 4L→4A).

**Figure 7:**
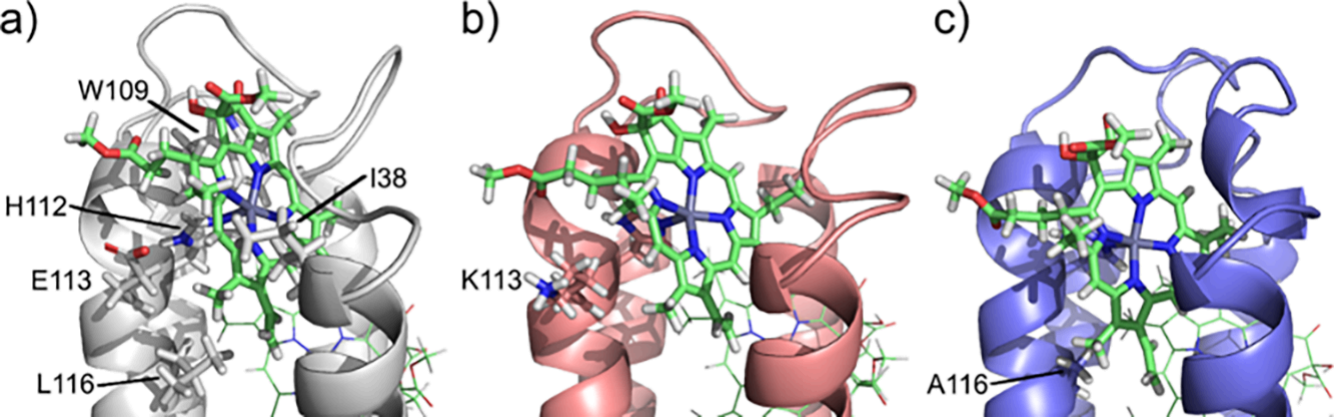
Main structural features in the chromophore binding pocket for (a) BT6, (b) BT6–4E→4K, and (c) BT6–4L→4A, as obtained from MD simulations. The images show the chromophore bound to H112 as an example, and correspond to representative structures obtained from a clustering analysis.

To calculate binding energies and compare them with previous results, we applied the QM/MM-PBSA method to the MD trajectories. While BT6 shows a total binding energy between the protein and chromophores of −203.7 kcal mol^–1^, in the mutants the value increases to −205.7 and −206.2 kcal mol^–1^ for 4E→4K and 4L→4A (respectively). This translates into slightly stronger binding energies by 2.0 and 2.5 kcal mol^–1^ (respectively), explaining why these two proteins can bind four chromophores despite the base design not being able to (due to unfavorable competition with chromophore aggregation).

We next analyzed the source of these improvements, by decomposing the changes on a per residue basis. Figure S18 shows that, for 4E→4K, the new K residues are the ones that contribute the most to increasing the binding energy, in line with the design hypothesis, based on the favorable electrostatic interactions between (positively charged) K and the (O-rich) polar side of the chromophore. On the other hand, as seen in Figure S19, for 4L→4A the effect of the mutations is less direct, as the contribution of the mutated residues is actually detrimental (which can be explained by the van der Waals interactions lost when swapping L by A). However, when considering the protein as a whole, the 4L→4A mutations do favor the binding. Decomposing the change in binding energy not by residue but by energy term (Table S2) confirms that the mutation is beneficial across the different components, with the largest contribution coming from the sum of the electrostatic interactions and polar solvation terms. This data suggests that, by reducing steric hindrance (replacing bulky L by smaller A), the chromophores can find a more accommodating binding pocket in this mutant.

Structural analyses confirm the more favorable pocket notion (Figure 8 and Table S3). When considering radii of gyration, both 4E→4K and 4L→4A show a smaller average value than BT6, with close-to-unimodal distributions centered at lower radii (Figure 8). The solvent accessible surface area shows a similar trend, decreasing in the mutants with respect to BT6 (more so in 4E→4K than in 4L→4A). All together, this indicates a more compact structure and a more deeply buried chromophore for both mutants. In addition, the average number of hydrogen bonds between the chromophores and the protein is larger for the mutants than for BT6 (Table S3). The mode value is 1 hydrogen bond (Figure 8); considering there are four chromophores per protein, this effectively indicates no permanent hydrogen bonds are established between the two. In any case, the distribution of values is skewed toward 0 for BT6, while higher numbers are more likely for the mutants, supporting the idea that the mutations help achieve a stronger interaction with the chromophore.

**Figure 8:**
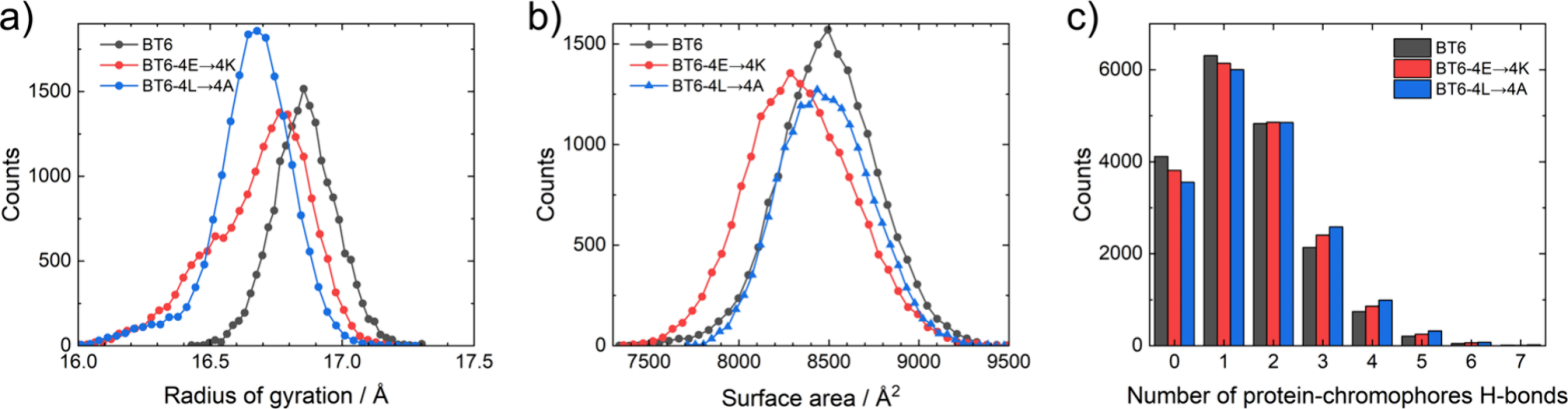
Histograms from structural metrics obtained from MD simulations of BT6, and the 4E→4K and 4L→4A mutants: (a) radius of gyration, (b) solvent-accessible surface area, and (c) number of H-bonds between the protein and the chromophores.

To assess the electronic structure of the complexes, we performed Born – Oppenheimer (BO) QM/MM MD simulations as continuation of the classical MDs, to obtain a set of high-quality chromophore geometries for each protein matrix (BT6, and the 4E→4K and 4L→4A mutants).^61^ After taking snapshots from these BOMD trajectories, we performed TD-DFT calculations at the QM/MMPol (polarizable MM) level,^62^ and applied state-specific corrections to the CT states energies.^63,64^ This state-of-the-art methodology allows to precisely capture the effect of the environment on the QM region, given that it is considered as polarizable, and thus reacts to the charges within the QM region. In addition, the state-specific corrections go beyond the linear response scheme and account for the polarization response of the environment to the new excited state density, and are particularly relevant for CT transitions as they depend on the change in permanent dipole moment from the ground state to the excited state.^63,65^

The results of these calculations are shown in Table 2. When considering ZnP homodimers (as in the experiments), we find similar results in the three complexes, pointing to a minimal influence from the protein matrix. That is, the Q_*y*_ state is between 1.96 and 1.99 eV, and the lowest-lying CT state is between 2.44 and 2.46 eV for all three systems, yielding an energy difference of 0.4 – 0.5 eV between these two states. The Q_*y*_ state shows however some mixing with the CT, as we find small contributions of CT-type single excitations beyond the main HOMO → LUMO contribution. These findings confirm the insights obtained from experiments: when ZnP homodimers are created, CT states are close to the Q_*y*_ states, with an energy difference conducive to a partial admixture of both states. This manifests itself by an increase in the dipole moment of the Q_*y*_ state upon dimer formation (Table 1), and by creating an additional decay channel related to charge separation and recombination.

**Table 2:** Excited States Energies, Obtained as Averages from QM/MMPol TD-DFT Calculations on Snapshots from BOMD Simulations

	**BT6 + ZnP homodimer**	**BT6 + ZnP/H2ZnP**	**BT6–4E→4K + ZnP homodimer**	**BT6–4L→4A + ZnP homodimer**
**Q**_*y*_ **energy [eV]**	1.99	2.02	1.98	1.96
**First CT energy [eV]**	2.44	1.98	2.46	2.46
**Energy difference [eV]**	+0.44	–0.04	+0.48	+0.50

We considered as well a system with asymmetric CT, replacing one of the ZnP chromophores by a derivative with a more favorable one-electron oxidation potential. Specifically, in this derivative (termed here H2-ZnP), the keto group in the fifth porphyrin ring is reduced to a methylene group, making this molecule more easily oxidizable.^66^ This has a direct impact on the CT state position (Table 2): now the first CT state (H2-ZnP → ZnP) is located at approximately the same energy level than the Q_*y*_ state, strongly favoring mixing between these two states. This result confirms the proposal that the CT states can be strongly stabilized by helping electron transfer between the chromophores; in this case, by favoring the one-electron oxidation of one of them.

## Conclusions

This study demonstrates the potential of de novo designed proteins to emulate natural photosynthetic protein functionalities through chromophore binding site engineering. By introducing excitonically coupled ZnP dimers, the presence of excited states with CT character is favored, which implies a significant alteration in the photophysical behavior of the assemblies. The mutants 4E→4K and 4L→4A revealed pronounced dipole moment changes (i.e., higher CT characters than the monomeric 2H→2A) upon excitation on account of the new electronic states derived from the dimerization process, with changes in dipole moments of 1.17 *Df* ^*1–*^/1.18 *Df* ^*1–*^ (high exciton) and 1.79 *Df* ^*1–*^/1.66 *Df* ^*1–*^ (low exciton) in 4E→4K and 4L→4A, respectively. This is double (high exciton) and triple (low exciton) of the value found for the benchmark mutant 2H→2A.

BB-TAS experiments with fs resolution allowed us to identify the changes on excited state dynamics upon incorporation of excitonically coupled ZnP dimers into the proteins with respect to the monomer case. Coupling between excitons and CT states results in additional nonradiative deactivation channels and short-lived charge separation. However, the role of ISC to the triplet manifold and phosphorescence was not explored explicitly and, consequently, further research on radiative relaxation pathways, in addition to temperature dependence studies that might identify which processes are thermally activated, are required in order to obtain a more concise picture of the excited state dynamics.

For a CT state to be energetically favored, the redox properties of the chromophores forming the dimer must favor electron transfer. The ZnP homodimer is in principle symmetric; charge transfer in such systems is commonly termed “symmetry breaking charge transfer”.^67^ Hence, future work on protein designs could focus on the construction of heterodimers as strategy to stabilize CT states. Aside from creating heterodimers, another possibility is the construction of proteins with binding pockets providing a different environment for each ZnP molecule in the dimer, creating asymmetric ZnP homodimers with the intention of fine-tuning the properties of CT states.

In summary, mixing of excitons and CT states, fast relaxation dynamics along the nuclear coordinate, and charge-separated states are key features for efficient light-harvesting and energy conversion processes, as observed in natural photosynthesis. Therefore, our findings highlight the tunability of synthetic protein scaffolds to optimize cofactor interactions and control their electronic properties, paving the way for advanced applications in bioinspired energy systems and photonic devices.

## Experimental Procedures

### Samples Preparation

Protein synthesis was performed via bacterial expression, as described in detail in a previous article.^27^ To synthesize the chromophore, pheophorbide *a* (Frontier Scientific) diastereoisomers were reacted with 5 mol equiv of ZnOAc_2_, as described in the literature.^19,68^ ZnP purity was monitored by High-Performance Liquid Chromatography (HPLC) and also by Ultra-Performance Liquid Chromatography coupled to High Resolution Mass Spectrometry (UPLC-HRMS).

Chromophore–protein complexes were initially prepared at a 5.0 μM protein concentration (estimated according to the absorption spectrum profile relative to the four tryptophan residues contained in each protein, ε_*280 nm*_ = 4 × 5625 L mol^–1^ cm^–1^ = 22500 L mol^–1^ cm^–1^) using a chromophore:protein concentration ratio of 2 for the 2H→2A mutant or 4 for the BT6–4E→4K and BT6–4L→4A mutants, incubated at 4 °C for 24 h, and then ultrafiltrated with a concentrator tube (VivaSpin, 3 kDa mass cutoff) until reaching a ≈1000 μM protein concentration. Subsequent dilutions were made based on the required optical density (OD) for each spectroscopic technique.

### Steady-State Measurements

Absorption and Stark spectra were recorded at 77 K using a liquid nitrogen cryostat (OptistatDN2, Oxford Instruments) in a home-built setup (see scheme in Figure S15). Light was generated with an Oriel TLS130B system, consisting of a 150 W xenon lamp powered by an OPS-A150 power supply, and a Cornerstone 130B monochromator (1/8m, Newport). The light output was horizontally polarized by a Glan-Thompson polarizer (10GT04, Newport). A silicon photodiode detector (DET-L-SIUV-R-C, Oriel) monitored the light intensity after propagating through the sample. This signal was fed into a lock-in amplifier (SR850, Stanford Research Systems). For absorption measurements, light intensity was modulated with an optical chopper (SR540, Stanford Research Systems) set at 333 Hz. For Stark measurements, a sine function with a frequency of 333 Hz was generated with the lock-in, fed into a high-voltage amplifier (10/10B-HS, Trek) that multiplied the voltage 1000-fold, and then applied to the Stark cell via a custom-made metallic rod. The Stark signal was detected at the second harmonic of the sine function frequency. Data acquisition was done through a customized LabVIEW (National Instruments) virtual instrument script that interfaced the lock-in amplifier, monochromator and detector.

The OD of the samples in the Stark cell (thickness: 100 μm as determined from interference fringes)^69^ was between 0.3 and 0.9 at the Q*_*y*_* band maximum (≈668 nm). The Stark cell was immersed in liquid nitrogen in the cryostat and rotated 45° with respect to the propagation direction of the horizontally polarized light, to set the angle between the electric field component of the light and the externally applied electric field to the magic angle (resulting when taking refraction at air-glass and glass-sample interfaces into account, 54.7°). The Stark spectra were recorded at an externally applied electric field strength of 2.2 · 10^5^ V cm^–1^ at a spectral resolution of 3 nm. Absorption spectra at room temperature and at 77 K were also taken in a Shimadzu UV–vis 2600i spectrophotometer. The spectra from the samples at 77K were scaled using the spectra measured in the Stark setup as a reference.

The Stark and absorbance spectra were fitted simultaneously using a nonlinear least-squares fitting program, written in Python within a JupyterLab notebook. The absorption spectra were fitted with a linear combination of several Gaussian functions, while the Stark spectra were fitted with the zeroth, first and second derivatives of these functions. The code is available at https://github.com/marianoastor/lipstark.

Fluorescence spectra were taken on a Fluorolog Horiba Jobin Yvon with a 10 mm quartz cuvette and in the front face configuration. The samples OD were maintained below 0.3 to minimize the inner filter effect.

### Time-Resolved Measurements

Ultrashort laser pulses were generated in a commercial Light Conversion Pharos laser system, which provides 200 fs pulses centered at 1030 nm with a repetition rate of 1 kHz. The fundamental output was directed into a home-built noncollinear optical parametric amplifier (NOPA) that permitted, after a compression stage involving chirped mirrors and a prism compressor, the generation of sub-15 fs pulses centered at 700 nm with a ∼125 nm bandwidth (full width at half-maximum, fwhm) for broadband transient absorption spectroscopy (BB-TAS) experiments. The temporal characterization of the pulses was conducted using a second harmonic frequency-resolved optical gating autocorrelator, as shown in Figure S16. The NOPA output was split into identical pump and probe beams, where usage of a linear translation stage (Newport XML210-S) enabled the control of the pump–probe time-delay up to 1 ns. The relative polarization between the pump and the probe pulses was set to either parallel or magic angle configuration and the pump beam was modulated at half the frequency of the laser repetition rate by a phased-locked optical chopper (Newport 3502) to obtain TA spectra in shot-to-shot subtraction. The samples were measured at room temperature in a 0.2 mm path length fused silica cuvette with an OD around 0.7 at the maximum of the Q*_*y*_* band. The excitation pulse energy was kept in the 3–5 nJ/pulse range to avoid annihilation effects (see Figure S17 for the power dependence measurements used to set the maximum pulse power). The probe beam transmitted through the sample was detected by a spectrometer (Teledyne Princeton Instruments Acton SP2300i) with a CCD camera (Teledyne PIXIS:100BR_eXcelon). Data collection and processing were carried out in a custom-made MATLAB script.

In order to extract the temporal and spectral information contained in the recorded data, first, the static scatter contributions were eliminated by subtracting a spectrum collected at negative time-delay. Then, using the open-source Glotaran software,^70,71^ the spectral chirp was corrected and a global fitting procedure was employed to model the time-traces at all the probe wavelengths with a set of exponential time-components (τ_*i*_) convoluted with a Gaussian instrument response function (IRF) of 45–80 fs (fwhm).

To complement the interpretation of BB-TAS data, the fluorescence decays of the complexes were obtained using a time-correlated single-photon counting (TCSPC) setup (Edinburgh Analytical Instruments LifeSpecII), with a pulsed diode operating at 405 nm excitation wavelength (model EPL-405, pulse width of 75 ps). The IRF was recorded using a Ludox sample. The emission decays were fitted as exponential functions using the software FAST. For all fluorescence decays, at least 5000 counts in the peak channel were accumulated for lifetime determination and a goodness-of-fit parameter χ^2^ was extracted from the software analysis. Good fits were assumed when χ^2^ < 1.200, along with visual inspection of the residuals of the fitted function to the data.

### Computational Modeling

All molecular dynamics (MD) simulations were performed with Amber22,^72^ using the ff14SB force field^73^ for proteins, TIP3P^74^ for water, and GAFF plus MCPB.py-derived parameters^75^ for the chromophores. Protonation states were determined with the H++ Web server at pH 9 (using default parameters).^76–78^ All residues were in their standard protonation state for this pH. Topology and geometry files were generated with LEaP, using an isometric truncated-octahedron shape for the periodic box, with a minimum distance between the protein and the edges of the box of 1.5 nm. Protein charges were neutralized with Na^+^ ions, and then NaCl was added to reach an ionic strength of ∼150 mM (as in experiments). The parametrization of the chromophores, together with the procedure to generate holo structures, is described in detail in previous work.^27^ The structures for the mutated proteins were generated using the Mutagenesis function within PyMOL,^79^ using previous BT6 simulations as the starting point.

Minimization and initial equilibration steps used the protocol of Roe.^80^ Briefly, it consists of nine sequential energy minimizations and short MD runs, which sum 4000 steps of minimization and 40000 MD steps (totalling 30 ps), followed by a final MD equilibration (500000 steps, 1000 ps). Then, production runs were done in the NPT ensemble at 300.0 K, with a time step of 2 fs, and constraining bonds involving hydrogen atoms via the SHAKE algorithm. Constant temperature and pressure were ensured with the Langevin thermostat (collision frequency: 2 ps^–1^) and Monte Carlo barostat, respectively. Long-range electrostatics were considered via the Particle Mesh Ewald (PME) model, setting the direct space sum cutoff to 1.0 nm. The first 80 ns were discarded (considered as equilibration), and the following time up to 1 μs was taken as production runs for each of four replicas performed on each system.

Representative structures from these runs were obtained from a clustering analysis, employing cpptraj^81^ and the hierarchical agglomerative approach. Clustering was done until the minimum distance between clusters was greater than 0.5 nm, and/or 5 clusters remained.

Binding energies were determined with the QM/Molecular Mechanics Generalized Born Surface Area (MMGBSA) method, using the MMPBSA.py module from AMBER,^82^ and the one-trajectory approach. Since the chromophore is covalently attached to the protein, to capture the stabilization from the Zn – N bond creation, it was necessary to include a QM region. It contained the chromophores and the histidine amino acids they were bound to, and was modeled with the PM6 ^83^ Hamiltonian. Solvation contributions were calculated with the Hawkins, Cramer and Truhlar model,^84^ using default parameters.

For the excited states calculations, we performed 1 ps QM/MM Born–Oppenheimer molecular dynamics (BOMD) simulations, describing one chromophore dimer and the two bound His residues in the QM region at the B3LYP/6–31G level of theory, and using the classical MDs as starting points. Gaussian 16 ^85^ was used as the QM engine. We then extracted 20 frames from the BOMD trajectory and performed single point TD-DFT excited state calculations using the polarizable embedding QM/MMPol model for dimer and monomer systems of the chromophore pair.^86,87^ We computed the 10 first excited states, describing the chromophores and His residues at the TD-CAM-B3LYP/6–31G­(d) level of theory and the protein+water environment using the amber pol12 AL polarizable force field,^88,89^ with charges for water derived from a previous work.^90^ The MMPol chromophores were also described using Amber pol12 AL with polarization-consistent ESP charges computed at the B3LYP/aug-cc-pVTZ level of theory. Explicit polarization was limited to residues within a cutoff radius of 12 Å from the QM heavy atoms (MMPol region), whereas residues up to a 25 Å were also included but adopting the additive force field (MM region). These calculations were performed using a locally modified development version of Gaussian.^91^

All data from molecular dynamics simulations has been deposited in the ioChem-BD database^92^ and is accessible through the doi:10.19061/iochem-bd-6–608.

## Supplementary Material



## Notes

Stark spectra and scheme for instrument setup; chromophore characterization; BB-TA and TCSPC data; supporting computational data (PDF)

## References

[1] Scholes G. D., Fleming G. R., Olaya-Castro A., van Grondelle R. (2011). Lessons from
Nature about Solar Light Harvesting. Nat. Chem..

[2] Blankenship, R. E. The Basic Principles of Photosynthetic Energy Storage. In Molecular Mechanisms of Photosynthesis; Wiley: 2002; pp 1–10. 10.1002/9780470758472.ch1.

[3] Wraight C. A., Clayton R. K. (1974). The Absolute Quantum
Efficiency of Bacteriochlorophyll
Photooxidation in Reaction Centres of Rhodopseudomonas Spheroides. Biochim. Biophys. Acta, Bioenerg..

[4] van
Grondelle R., Dekker J. P., Gillbro T., Sundstrom V. (1994). Energy Transfer
and Trapping in Photosynthesis. Biochimica et
Biophysica Acta (BBA) - Bioenergetics.

[5] Ford R. C. (1992). Photosynthetic
Membrane Proteins. Curr. Opin. Struct. Biol..

[6] Wang L., Roth J. S., Han X., Evans S. D. (2015). Photosynthetic Proteins
in Supported Lipid Bilayers: Towards a Biokleptic Approach for Energy
Capture. Small.

[7] Dekker J. P., Boekema E. J. (2005). Supramolecular Organization
of Thylakoid Membrane Proteins
in Green Plants. Biochimica et Biophysica Acta
(BBA) - Bioenergetics.

[8] Blankenship, R. E. Photosynthetic Pigments: Structure and Spectroscopy. In Molecular Mechanisms of Photosynthesis; Wiley: 2002; pp 42–60. 10.1002/9780470758472.ch4.

[9] Romero E., Diner B. A., Nixon P. J., Coleman W. J., Dekker J. P., van Grondelle R. (2012). Mixed Exciton–Charge-Transfer
States in Photosystem
II: Stark Spectroscopy on Site-Directed Mutants. Biophys. J..

[10] Fujimoto K. J., Tsuji R., Wang-Otomo Z.-Y., Yanai T. (2024). Prominent Role of Charge
Transfer in the Spectral Tuning of Photosynthetic Light-Harvesting
I Complex. ACS Phys. Chem. Au.

[11] Treynor T. P., Andrews S. S., Boxer S. G. (2003). Intervalence
Band Stark Effect of
the Special Pair Radical Cation in Bacterial Photosynthetic Reaction
Centers. J. Phys. Chem. B.

[12] Capone M., Sirohiwal A., Aschi M., Pantazis D. A., Daidone I. (2023). Alternative
Fast and Slow Primary Charge-Separation Pathways in Photosystem II. Angew. Chem., Int. Ed..

[13] Hobbs C. J., Roach N., Wagner P., van der Salm H., Barnsley J. E., Gordon K. C., Kodali G., Moser C. C., Dutton P. L., Wagner K., Officer D. L. (2020). Emulating
Photosynthetic
Processes with Light Harvesting Synthetic Protein (Maquette) Assemblies
on Titanium Dioxide. Mater. Adv..

[14] Chang R., Zhao L., Xing R., Li J., Yan X. (2023). Functional
Chromopeptide Nanoarchitectonics: Molecular Design, Self-Assembly
and Biological Applications. Chem. Soc. Rev..

[15] Wei H., Min J., Wang Y., Shen Y., Du Y., Su R., Qi W. (2022). Bioinspired
Porphyrin–Peptide Supramolecular Assemblies and
Their Applications. J. Mater. Chem. B.

[16] Ennist N.
M., Wang S., Kennedy M. A., Curti M., Sutherland G. A., Vasilev C., Redler R. L., Maffeis V., Shareef S., Sica A. V., Hua A. S., Deshmukh A. P., Moyer A. P., Hicks D. R., Swartz A. Z., Cacho R. A., Novy N., Bera A. K., Kang A., Sankaran B., Johnson M. P., Phadkule A., Reppert M., Ekiert D., Bhabha G., Stewart L., Caram J. R., Stoddard B. L., Romero E., Hunter C. N., Baker D. (2024). De Novo Design of Proteins Housing
Excitonically Coupled Chlorophyll Special Pairs. Nat. Chem. Biol..

[17] Mancini J. A., Sheehan M., Kodali G., Chow B. Y., Bryant D. A., Dutton P. L., Moser C. C. (2018). De Novo Synthetic
Biliprotein Design,
Assembly and Excitation Energy Transfer. Journal
of The Royal Society Interface.

[18] Liu J., Zheng Q., Deng Y., Li Q., Kallenbach N. R., Lu M. (2007). Conformational Specificity
of the Lac Repressor Coiled-Coil Tetramerization
Domain. Biochemistry.

[19] Kodali G., Mancini J. A., Solomon L. A., Episova T. V., Roach N., Hobbs C. J., Wagner P., Mass O. A., Aravindu K., Barnsley J. E., Gordon K. C., Officer D. L., Dutton P. L., Moser C. C. (2017). Design and Engineering
of Water-Soluble Light-Harvesting
Protein Maquettes. Chem. Sci..

[20] Fry H. C., Lehmann A., Sinks L. E., Asselberghs I., Tronin A., Krishnan V., Blasie J. K., Clays K., DeGrado W. F., Saven J. G., Therien M. J. (2013). Computational
de
Novo Design and Characterization of a Protein That Selectively Binds
a Highly Hyperpolarizable Abiological Chromophore. J. Am. Chem. Soc..

[21] Koo J., Park J., Tronin A., Zhang R., Krishnan V., Strzalka J., Kuzmenko I., Fry H. C., Therien M. J., Blasie J. K. (2012). Acentric 2-D Ensembles
of D-Br-A Electron-Transfer
Chromophores via Vectorial Orientation within Amphiphilic n-Helix
Bundle Peptides for Photovoltaic Device Applications. Langmuir.

[22] Strzalka J., Xu T., Tronin A., Wu S. P., Miloradovic I., Kuzmenko I., Gog T., Therien M. J., Blasie J. K. (2006). Structural
Studies of Amphiphilic 4-Helix Bundle Peptides Incorporating Designed
Extended Chromophores for Nonlinear Optical Biomolecular Materials. Nano Lett..

[23] Mann S. I., Nayak A., Gassner G. T., Therien M. J., DeGrado W. F. (2021). De Novo
Design, Solution Characterization, and Crystallographic Structure
of an Abiological Mn–Porphyrin-Binding Protein Capable of Stabilizing
a Mn­(V) Species. J. Am. Chem. Soc..

[24] Cohen-Ofri I., van Gastel M., Grzyb J., Brandis A., Pinkas I., Lubitz W., Noy D. (2011). Zinc-Bacteriochlorophyllide Dimers
in de Novo Designed Four-Helix Bundle Proteins. A Model System for
Natural Light Energy Harvesting and Dissipation. J. Am. Chem. Soc..

[25] Ennist N. M., Zhao Z., Stayrook S. E., Discher B. M., Dutton P. L., Moser C. C. (2022). De Novo Protein
Design of Photochemical Reaction Centers. Nat.
Commun..

[26] Farid T. A., Kodali G., Solomon L. A., Lichtenstein B. R., Sheehan M. M., Fry B. A., Bialas C., Ennist N. M., Siedlecki J. A., Zhao Z., Stetz M. A., Valentine K. G., Anderson J. L. R., Wand A. J., Discher B. M., Moser C. C., Dutton P. L. (2013). Elementary Tetrahelical Protein Design for Diverse
Oxidoreductase Functions. Nat. Chem. Biol..

[27] Curti M., Maffeis V., Teixeira Alves Duarte L. G., Shareef S., Hallado L. X., Curutchet C., Romero E. (2023). Engineering Excitonically
Coupled Dimers in an Artificial Protein for Light Harvesting via Computational
Modeling. Protein Sci..

[28] Krawczyk S. (1991). Electrochromism
of Chlorophyll a Monomer and Special Pair Dimer. Biochimica et Biophysica Acta (BBA) - Bioenergetics.

[29] Romero E., Mozzo M., van Stokkum I. H. M., Dekker J. P., van Grondelle R., Croce R. (2009). The Origin of the Low-Energy Form of Photosystem I Light-Harvesting
Complex Lhca4: Mixing of the Lowest Exciton with a Charge-Transfer
State. Biophys. J..

[30] Bublitz G. U., Boxer S. G. (1997). STARK SPECTROSCOPY:
Applications in Chemistry, Biology,
and Materials Science. Annu. Rev. Phys. Chem..

[31] Wahadoszamen Md., Margalit I., Ara A. M., van Grondelle R., Noy D. (2014). The Role of Charge-Transfer States
in Energy Transfer and Dissipation
within Natural and Artificial Bacteriochlorophyll Proteins. Nat. Commun..

[32] Renge I., Mauring K. (2013). Spectral Shift Mechanisms of Chlorophylls in Liquids
and Proteins. Spectrochimica Acta Part A: Molecular
and Biomolecular Spectroscopy.

[33] Moretti L., Kudisch B., Terazono Y., Moore A. L., Moore T. A., Gust D., Cerullo G., Scholes G. D., Maiuri M. (2020). Ultrafast
Dynamics of Nonrigid Zinc-Porphyrin Arrays Mimicking the Photosynthetic
“Special Pair”. J. Phys. Chem.
Lett..

[34] Kumble R., Palese S., Lin V. S.-Y., Therien M. J., Hochstrasser R. M. (1998). Ultrafast
Dynamics of Highly Conjugated Porphyrin Arrays. J. Am. Chem. Soc..

[35] Gurzadyan G. G., Tran-Thi T.-H., Gustavsson T. (1998). Time-Resolved
Fluorescence Spectroscopy
of High-Lying Electronic States of Zn-Tetraphenylporphyrin. J. Chem. Phys..

[36] Baskin J. S., Yu H.-Z., Zewail A. H. (2002). Ultrafast
Dynamics of Porphyrins
in the Condensed Phase: I. Free Base Tetraphenylporphyrin. J. Phys. Chem. A.

[37] Yu H.-Z., Baskin J. S., Zewail A. H. (2002). Ultrafast
Dynamics of Porphyrins
in the Condensed Phase: II. Zinc Tetraphenylporphyrin. J. Phys. Chem. A.

[38] Enescu M., Steenkeste K., Tfibel F., Fontaine-Aupart M.-P. (2002). Femtosecond
Relaxation Processes from Upper Excited States of Tetrakis­(N-Methyl-4-Pyridyl)­Porphyrins
Studied by Transient Absorption Spectroscopy. Phys. Chem. Chem. Phys..

[39] Yoon M.-C., Jeong D. H., Cho S., Kim D., Rhee H., Joo T. (2003). Ultrafast Transient Dynamics of Zn­(II)
Porphyrins: Observation of
Vibrational Coherence by Controlling Chirp of Femtosecond Pulses. J. Chem. Phys..

[40] Collini E., Ferrante C., Bozio R. (2007). Influence
of Excitonic Interactions
on the Transient Absorption and Two-Photon Absorption Spectra of Porphyrin
J-Aggregates in the NIR Region. J. Phys. Chem.
C.

[41] Kullmann M., Hipke A., Nuernberger P., Bruhn T., Götz D. C. G., Sekita M., Guldi D. M., Bringmann G., Brixner T. (2012). Ultrafast Exciton Dynamics after Soret- or Q-Band Excitation
of a Directly β,Β′-Linked Bisporphyrin. Phys. Chem. Chem. Phys..

[42] Bräm O., Cannizzo A., Chergui M. (2019). Ultrafast
Broadband Fluorescence
Up-Conversion Study of the Electronic Relaxation of Metalloporphyrins. J. Phys. Chem. A.

[43] Petropoulos V., Rukin P. S., Quintela F., Russo M., Moretti L., Moore A., Moore T., Gust D., Prezzi D., Scholes G. D., Molinari E., Cerullo G., Troiani F., Rozzi C. A., Maiuri M. (2024). Vibronic Coupling
Drives the Ultrafast
Internal Conversion in a Functionalized Free-Base Porphyrin. J. Phys. Chem. Lett..

[44] Van
Stokkum I. H. M., Larsen D. S., Van Grondelle R. (2004). Global and
Target Analysis of Time-Resolved Spectra. Biochimica
et Biophysica Acta (BBA)-Bioenergetics.

[45] Ohno O., Kaizu Y., Kobayashi H. (1985). Luminescence
of Some Metalloporphins
Including the Complexes of the IIIb Metal Group. J. Chem. Phys..

[46] Mennenga A., Gärtner W., Lubitz W., Görner H. (2006). Effects of
Noncovalently Bound Quinones on the Ground and Triplet States of Zinc
Chlorins in Solution and Bound to de Novo Synthesized Peptides. Phys. Chem. Chem. Phys..

[47] Arshad A., Castellano F. N. (2024). Homomolecular
Triplet–Triplet Annihilation in
Metalloporphyrin Photosensitizers. J. Phys.
Chem. A.

[48] Romero E., Van Stokkum I. H. M., Novoderezhkin V. I., Dekker J. P., Van Grondelle R. (2010). Two Different
Charge Separation Pathways in Photosystem II. Biochemistry.

[49] Akhtar P., Sipka G., Han W., Li X., Han G., Shen J.-R., Garab G., Tan H.-S., Lambrev P. H. (2022). Ultrafast
Excitation Quenching by the Oxidized Photosystem II Reaction Center. J. Chem. Phys..

[50] Müller M. G., Slavov C., Luthra R., Redding K. E., Holzwarth A. R. (2010). Independent
Initiation of Primary Electron Transfer in the Two Branches of the
Photosystem I Reaction Center. Proc. Natl. Acad.
Sci. U.S.A..

[51] Van
Stokkum I. H. M., Müller M. G., Holzwarth A. R. (2024). Energy
Transfer and Radical-Pair Dynamics in Photosystem I with Different
Red Chlorophyll a Pigments. IJMS.

[52] Giera W., Ramesh V. M., Webber A. N., van Stokkum I., van Grondelle R., Gibasiewicz K. (2010). Effect of
the P700 Pre-Oxidation
and Point Mutations near A0 on the Reversibility of the Primary Charge
Separation in Photosystem I from Chlamydomonas Reinhardtii. Biochimica et Biophysica Acta (BBA) - Bioenergetics.

[53] Russo M., Casazza A. P., Cerullo G., Santabarbara S., Maiuri M. (2022). Ultrafast Excited State Dynamics in the Monomeric and
Trimeric Photosystem I Core Complex of *Spirulina Platensis* Probed by Two-Dimensional Electronic Spectroscopy. J. Chem. Phys..

[54] Rodriguez J., Kirmaier C., Holten D. (1989). Optical Properties
of Metalloporphyrin
Excited States. J. Am. Chem. Soc..

[55] Pekkarinen L., Linschitz H. (1960). Studies on Metastable States of Porphyrins. II. Spectra
and Decay Kinetics of Tetraphenylporphine, Zinc Tetraphenylporphine
and Bacteriochlorophyll1. J. Am. Chem. Soc..

[56] Moravec D. B., Lovaasen B. M., Hopkins M. D. (2013). Near-Infrared Transient-Absorption
Spectroscopy of Zinc Tetraphenylporphyrin and Related Compounds. Observation
of Bands That Selectively Probe the S1 Excited State. J. Photochem. Photobiol., A.

[57] Gerola A. P., Tsubone T. M., Santana A., De Oliveira H. P. M., Hioka N., Caetano W. (2011). Properties of Chlorophyll
and Derivatives
in Homogeneous and Microheterogeneous Systems. J. Phys. Chem. B.

[58] Baskin J. S., Zewail A. H. (1994). Femtosecond Real-Time
Probing of Reactions. 15. Time-Dependent
Coherent Alignment. J. Phys. Chem..

[59] Imanbaew D., Gelin M. F., Riehn C. (2016). Rotational and Vibrational Dynamics
in the Excited Electronic State of Deprotonated and Protonated Fluorescein
Studied by Time-Resolved Photofragmentation in an Ion Trap. Struct. Dyn..

[60] Pereira M. A., Share P. E., Sarisky M. J., Hochstrasser R. M. (1991). Ultrafast
Rotational Dynamics of Electronically Excited Aniline Molecules in
Solution from Ultraviolet Femtosecond Fluorescence Anisotropies. J. Chem. Phys..

[61] Ozaydin B., Curutchet C. (2023). Unraveling
the Role of Thermal Fluctuations on the
Exciton Structure of the Cryptophyte PC612 and PC645 Photosynthetic
Antenna Complexes. Front. Mol. Biosci..

[62] Curutchet C., Kongsted J., Muñoz-Losa A., Hossein-Nejad H., Scholes G. D., Mennucci B. (2011). Photosynthetic Light-Harvesting
Is
Tuned by the Heterogeneous Polarizable Environment of the Protein. J. Am. Chem. Soc..

[63] Guareschi R., Valsson O., Curutchet C., Mennucci B., Filippi C. (2016). Electrostatic
versus Resonance Interactions in Photoreceptor Proteins: The Case
of Rhodopsin. J. Phys. Chem. Lett..

[64] Loco D., Polack Eí., Caprasecca S., Lagardère L., Lipparini F., Piquemal J.-P., Mennucci B. (2016). A QM/MM Approach Using
the AMOEBA Polarizable Embedding: From Ground State Energies to Electronic
Excitations. J. Chem. Theory Comput..

[65] Guido C. A., Chrayteh A., Scalmani G., Mennucci B., Jacquemin D. (2021). Simple Protocol
for Capturing Both Linear-Response and State-Specific Effects in Excited-State
Calculations with Continuum Solvation Models. J. Chem. Theory Comput..

[66] Sasaki S., Yoshizato M., Kunieda M., Tamiaki H. (2010). Cooperative C3- and
C13-Substituent Effects on Synthetic Chlorophyll Derivatives. Eur. J. Org. Chem..

[67] Hart S. M., Banal J. L., Castellanos M. A., Markova L., Vyborna Y., Gorman J., Häner R., Willard A. P., Bathe M., Schlau-Cohen G. S. (2022). Activating
Charge-Transfer State Formation in Strongly-Coupled
Dimers Using DNA Scaffolds. Chem. Sci..

[68] Hartwich G., Fiedor L., Simonin I., Cmiel E., Schäfer W., Noy D., Scherz A., Scheer H. (1998). Metal-Substituted Bacteriochlorophylls.
1. Preparation and Influence of Metal and Coordination on Spectra. J. Am. Chem. Soc..

[69] Huibers P. D. T., Shah D. O. (1997). Multispectral Determination
of Soap Film Thickness. Langmuir.

[70] Snellenburg J. J., Laptenok S., Seger R., Mullen K. M., van Stokkum I. H. (2012). Glotaran:
A Java-Based Graphical User Interface for the R Package TIMP. J. Stat. Software.

[71] van
Stokkum I. H. M., Weißenborn J., Weigand S., Snellenburg J. J. (2023). Pyglotaran:
A Lego-like Python Framework for Global and Target Analysis of Time-Resolved
Spectra. Photochemical & Photobiological
Sciences.

[72] Case, D. A. ; Walker, R. C. ; Cheatham, T. E. ; Simmerling, C. ; Roitberg, A. ; Merz, K. M. ; Luo, R. ; Darden, T. Amber 18; University of California: San Francisco, 2018, 1–923.

[73] Maier J. A., Martinez C., Kasavajhala K., Wickstrom L., Hauser K. E., Simmerling C. (2015). ff14SB: Improving
the Accuracy of
Protein Side Chain and Backbone Parameters from ff99SB. J. Chem. Theory Comput..

[74] Jorgensen W. L., Chandrasekhar J., Madura J. D., Impey R. W., Klein M. L. (1983). Comparison
of Simple Potential Functions for Simulating Liquid Water. J. Chem. Phys..

[75] Li P., Merz K. M. (2016). MCPB.Py: A Python Based Metal Center
Parameter Builder. J. Chem. Inf. Model..

[76] Myers J., Grothaus G., Narayanan S., Onufriev A. (2006). A Simple Clustering
Algorithm Can Be Accurate Enough for Use in Calculations of pKs in
Macromolecules. Proteins: Struct., Funct., Genet..

[77] Anandakrishnan R., Aguilar B., Onufriev A. V. (2012). H++ 3.0: Automating pK Prediction
and the Preparation of Biomolecular Structures for Atomistic Molecular
Modeling and Simulations. Nucleic Acids Res..

[78] Gordon J. C., Myers J. B., Folta T., Shoja V., Heath L. S., Onufriev A. (2005). H++: A Server for Estimating pKas and Adding Missing
Hydrogens to Macromolecules. Nucleic Acids Res..

[79] Schrödinger, L. L. C. ; DeLano, W. PyMOL, 2020.

[80] Roe D. R., Brooks B. R. (2020). A Protocol for Preparing
Explicitly Solvated Systems
for Stable Molecular Dynamics Simulations. J.
Chem. Phys..

[81] Roe D. R., Cheatham T. E. (2013). PTRAJ and CPPTRAJ: Software for Processing and Analysis
of Molecular Dynamics Trajectory Data. J. Chem.
Theory Comput..

[82] Miller B. R., McGee T. D., Swails J. M., Homeyer N., Gohlke H., Roitberg A. E. (2012). MMPBSA.Py: An Efficient Program for End-State Free
Energy Calculations. J. Chem. Theory Comput..

[83] Stewart J.
J. P. (2007). Optimization
of Parameters for Semiempirical Methods V: Modification of NDDO Approximations
and Application to 70 Elements. J. Mol. Model..

[84] Hawkins G. D., Cramer C. J., Truhlar D. G. (1996). Parametrized Models of Aqueous Free
Energies of Solvation Based on Pairwise Descreening of Solute Atomic
Charges from a Dielectric Medium. J. Phys. Chem..

[85] Frisch, M. J. ; Trucks, G. W. ; Schlegel, H. B. ; Scuseria, G. E. ; Robb, M. A. ; Cheeseman, J. R. ; Scalmani, G. ; Barone, V. ; Petersson, G. A. ; Nakatsuji, H. ; Li, X. ; Caricato, M. ; Marenich, A. V. ; Bloino, J. ; Janesko, B. G. ; Gomperts, R. ; Mennucci, B. ; Hratchian, H. P. ; Ortiz, J. V. ; Izmaylov, A. F. ; Sonnenberg, J. L. ; Williams; Ding, F. ; Lipparini, F. ; Egidi, F. ; Goings, J. ; Peng, B. ; Petrone, A. ; Henderson, T. ; Ranasinghe, D. ; Zakrzewski, V. G. ; Gao, J. ; Rega, N. ; Zheng, G. ; Liang, W. ; Hada, M. ; Ehara, M. ; Toyota, K. ; Fukuda, R. ; Hasegawa, J. ; Ishida, M. ; Nakajima, T. ; Honda, Y. ; Kitao, O. ; Nakai, H. ; Vreven, T. ; Throssell, K. ; Montgomery, J. A., Jr. ; Peralta, J. E. ; Ogliaro, F. ; Bearpark, M. J. ; Heyd, J. J. ; Brothers, E. N. ; Kudin, K. N. ; Staroverov, V. N. ; Keith, T. A. ; Kobayashi, R. ; Normand, J. ; Raghavachari, K. ; Rendell, A. P. ; Burant, J. C. ; Iyengar, S. S. ; Tomasi, J. ; Cossi, M. ; Millam, J. M. ; Klene, M. ; Adamo, C. ; Cammi, R. ; Ochterski, J. W. ; Martin, R. L. ; Morokuma, K. ; Farkas, O. ; Foresman, J. B. ; Fox, D. J. Gaussian 16 Rev. C.01, Gaussian Inc.: Wallingford CT, 2016.

[86] Curutchet C., Muñoz-Losa A., Monti S., Kongsted J., Scholes G. D., Mennucci B. (2009). Electronic Energy Transfer in Condensed Phase Studied
by a Polarizable QM/MM Model. J. Chem. Theory
Comput..

[87] Curutchet C., Mennucci B. (2017). Quantum Chemical Studies of Light Harvesting. Chem. Rev..

[88] Wang J., Cieplak P., Li J., Hou T., Luo R., Duan Y. (2011). Development of Polarizable Models for Molecular Mechanical
Calculations
I: Parameterization of Atomic Polarizability. J. Phys. Chem. B.

[89] Wang J., Cieplak P., Li J., Wang J., Cai Q., Hsieh M., Lei H., Luo R., Duan Y. (2011). Development
of Polarizable Models for Molecular Mechanical Calculations II: Induced
Dipole Models Significantly Improve Accuracy of Intermolecular Interaction
Energies. J. Phys. Chem. B.

[90] Corbella M., Cupellini L., Lipparini F., Scholes G. D., Curutchet C. (2019). Spectral Variability
in Phycocyanin Cryptophyte Antenna Complexes Is Controlled by Changes
in the α-Polypeptide Chains. ChemPhotoChem..

[91] Frisch, M. J. ; Trucks, G. W. ; Schlegel, H. B. ; Scuseria, G. E. ; Robb, M. A. ; Cheeseman, J. R. ; Scalmani, G. ; Barone, V. ; Petersson, G. A. ; Nakatsuji, H. ; Li, X. ; Caricato, M. ; Marenich, A. V. ; Bloino, J. ; Janesko, B. G. ; Gomperts, R. ; Mennucci, B. ; Hratchian, H. P. ; Ortiz, J. V. ; Izmaylov, A. F. ; Sonnenberg, J. L. ; Williams; Ding, F. ; Lipparini, F. ; Egidi, F. ; Goings, J. ; Peng, B. ; Petrone, A. ; Henderson, T. ; Ranasinghe, D. ; Zakrzewski, V. G. ; Gao, J. ; Rega, N. ; Zheng, G. ; Liang, W. ; Hada, M. ; Ehara, M. ; Toyota, K. ; Fukuda, R. ; Hasegawa, J. ; Ishida, M. ; Nakajima, T. ; Honda, Y. ; Kitao, O. ; Nakai, H. ; Vreven, T. ; Throssell, K. ; Montgomery, J. A., Jr. ; Peralta, J. E. ; Ogliaro, F. ; Bearpark, M. J. ; Heyd, J. J. ; Brothers, E. N. ; Kudin, K. N. ; Staroverov, V. N. ; Keith, T. A. ; Kobayashi, R. ; Normand, J. ; Raghavachari, K. ; Rendell, A. P. ; Burant, J. C. ; Iyengar, S. S. ; Tomasi, J. ; Cossi, M. ; Millam, J. M. ; Klene, M. ; Adamo, C. ; Cammi, R. ; Ochterski, J. W. ; Martin, R. L. ; Morokuma, K. ; Farkas, O. ; Foresman, J. B. ; Fox, D. J. Gaussian Development Version, Revision H.36, 2010.

[92] Aílvarez-Moreno M., de Graaf C., Loípez N., Maseras F., Poblet J. M., Bo C. (2015). Managing the Computational Chemistry Big Data Problem: The ioChem-BD
Platform. J. Chem. Inf. Model..

