# Supplementary material for: Unraveling Charge-Transfer States and Their Ultrafast Dynamics in Artificial Light-Harvesting Complexes
Source: ACS Phys Chem Au. 2026 Jan 12;6(2):286–300. doi: 10.1021/acsphyschemau.5c00098 (PMC13022723; doi:10.1021/acsphyschemau.5c00098)
Supplement: Supplementary file 1 [file pg5c00098_si_001.pdf]

Supporting Information

Unraveling Charge-Transfer States and their Ultrafast Dynamics in Artificial Light Harvesting Complexes

Luís Gustavo Teixeira Alves Duarte,[1] Iker Lamas,[1] Dominik Bäuerle,[1,2] Saeed Shareef,[1,2] Renato D. Cunha,[3,4,5] Carles Curutchet,[3,4] Mariano Curti,[3,4,] and Elisabet Romero[1,*]*

[1]Institute of Chemical Research of Catalonia (ICIQ-CERCA), Barcelona Institute of Science and Technology (BIST), Avda. Països Catalans 16, Tarragona 43007, Spain.

[2]Departament de Química Física i Inorgànica, Universitat Rovira i Virgili, C/Marcel·lí Domingo s/n, Tarragona 43007, Spain.

[3]Departament de Farmàcia i Tecnologia Farmacèutica, i Fisicoquímica, Facultat de Farmàcia i Ciències de l'Alimentació, Universitat de Barcelona (UB), Av. Joan XXIII 27-31, 08028, Barcelona, Spain

[4]Institut de Química Teòrica i Computacional (IQTC-UB), Universitat de Barcelona (UB), Barcelona, Spain

[5]Aix Marseille Univ, CNRS, ICR, 13397 Marseille, France

** Corresponding authors, emails: mcurti@ub.edu, eromero@iciq.es*

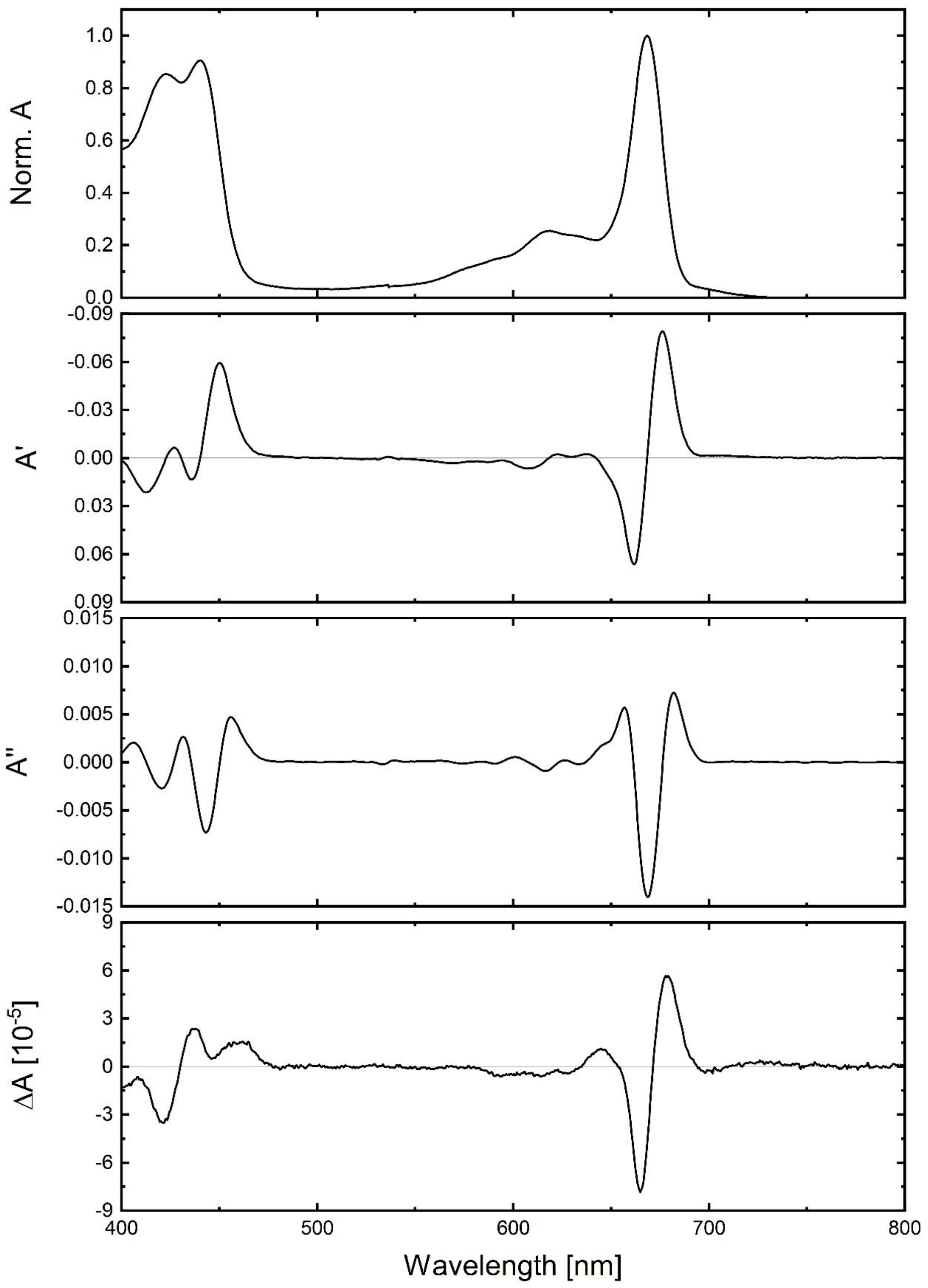

Figure S1. Absorption spectra at 77 K, its first and second derivatives, and Stark spectrum for 2H→2A.

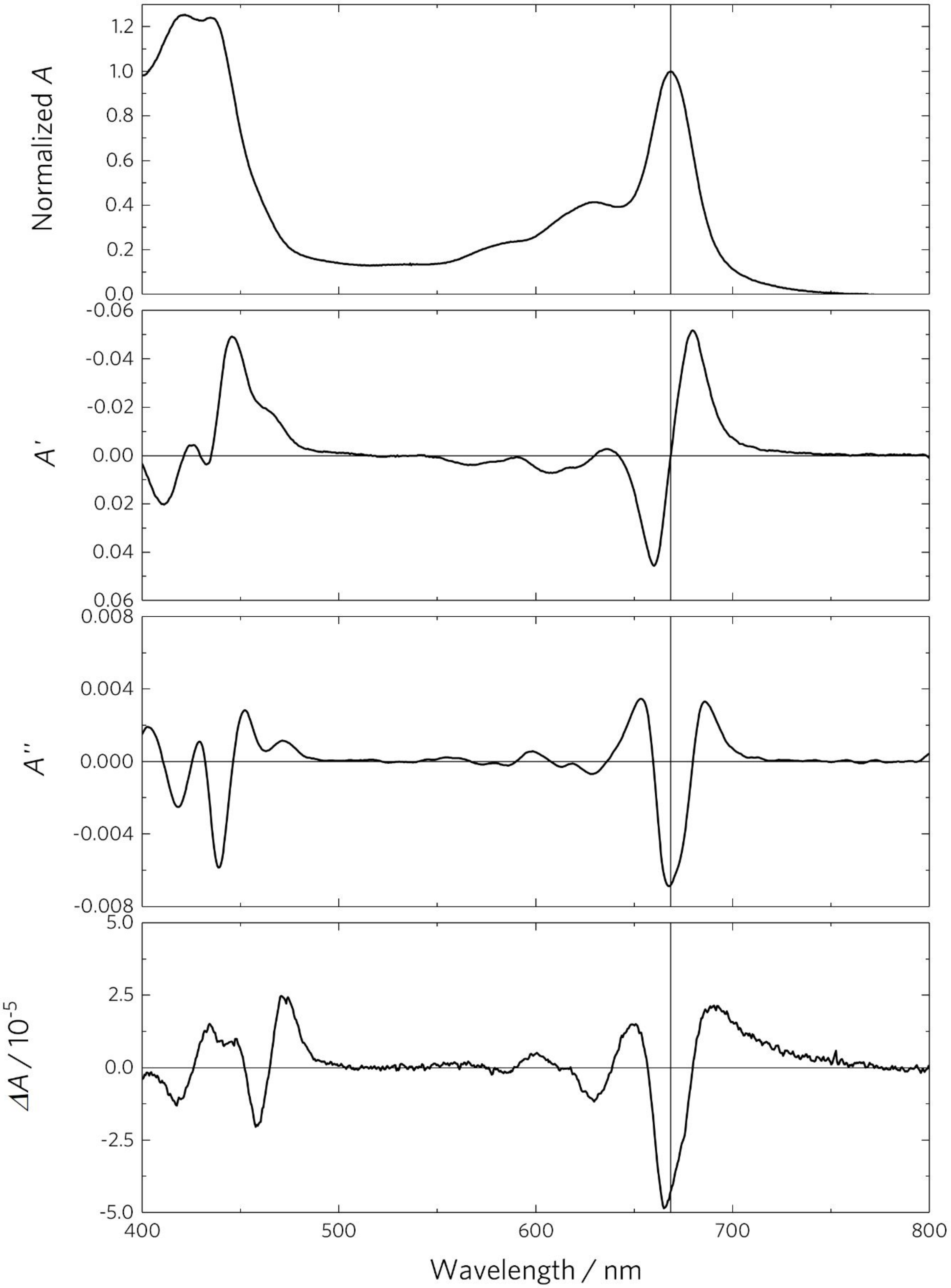

Figure S2. Absorption spectra at 77 K, its first and second derivatives, and Stark spectrum for 4E→4K.

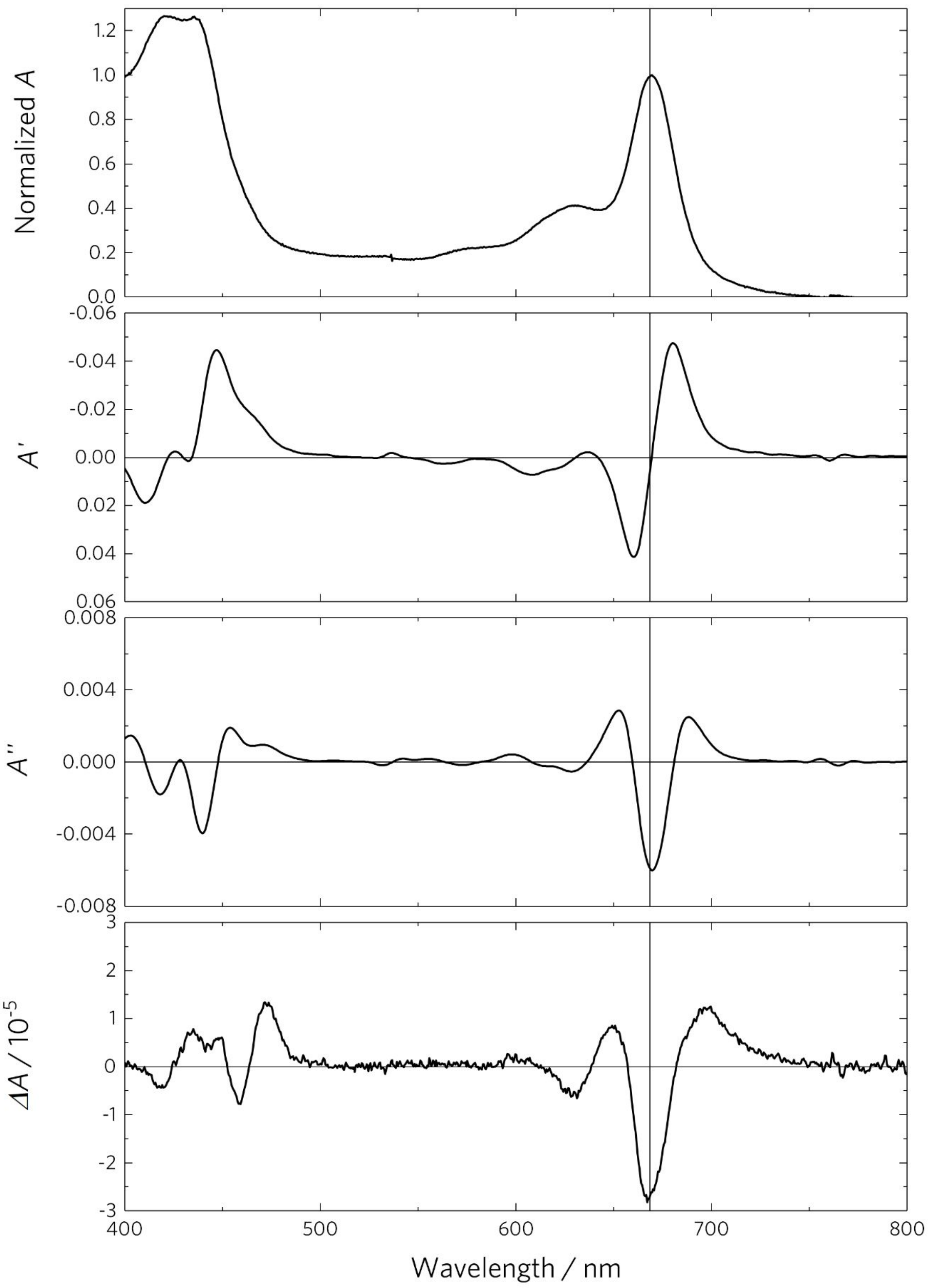

Figure S3. Absorption spectra at 77 K, its first and second derivatives, and Stark spectrum for 4L→4A.

Supplementary note 1: Chromophore purity

ZnP purity was checked by High-Performance Liquid Chromatography (HPCL, Agilient 1100 – MSD 6130, with a Diode Array Detector). The obtained chromatogram presented five components. The respective absorption spectra were determined throughout the purification procedure, revealing that the last component, which corresponds to 10% of the sample, has a slightly different spectrum with a red tail (Figure S7a and S7b). Subsequent analysis by Ultra-Performance Liquid Chromatography coupled to High Resolution Mass Spectrometry (UPLC-HRMS, Bruker Maxis Impact QqTOF) helped on the proposal of the molecular structures of each component found in the chromatogram, which correspond to ZnP diastereomers, a ZnP hydroxylated form, its dimethylated version and a product of a side reaction that induces formation of a lactone derivative (Figures S8 to S13). The minor structural variations of the first four components, away from the porphyrin ring, corroborate with the absorption spectra assignment.

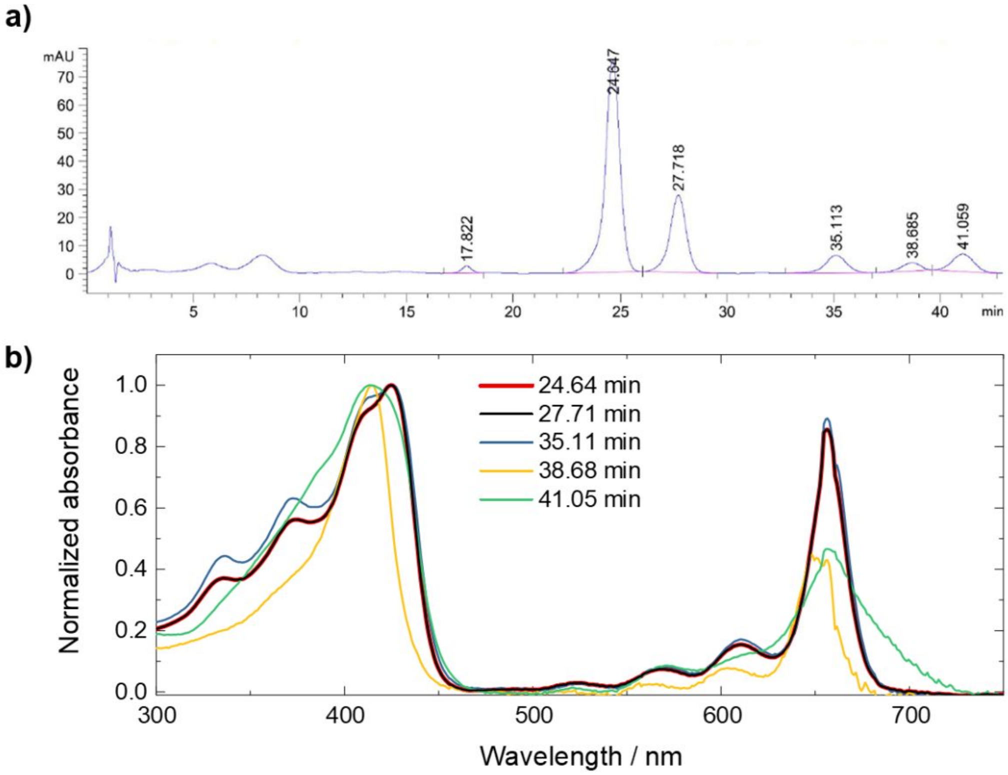

Figure S4. (a) ZnP chromatogram and (b) the respective absorption spectra of each one of the observed peaks. Numbers close to each peak indicates its retention time and relative area.

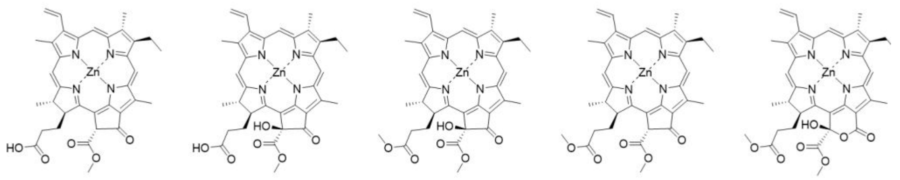

	24.64 min and 27.71 min	24.64 min	35.11 min	38.68 min	41.05 min
m/z experimental	653.1735	671.1804	685.1983	669.1668	701.1917
m/z calculated	653.1715	671.1848	685.1991	669.1705	701.1940

Figure S5. Molecular structures of ZnP observed by UPLC-HRMS. The listed retention times are the ones obtained from HPLC.

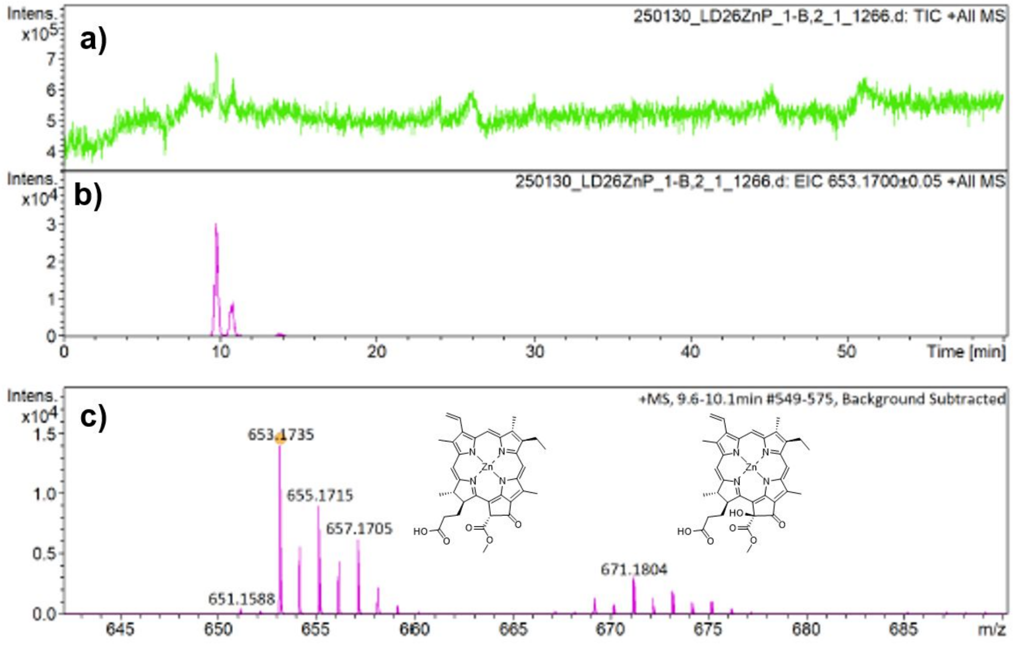

Figure S6. (a) Total Ion Current (TIC) and (b) chromatogram peak obtained by UPLC between 9.6 and 10.1 min with the respective (c) HRMS spectra.

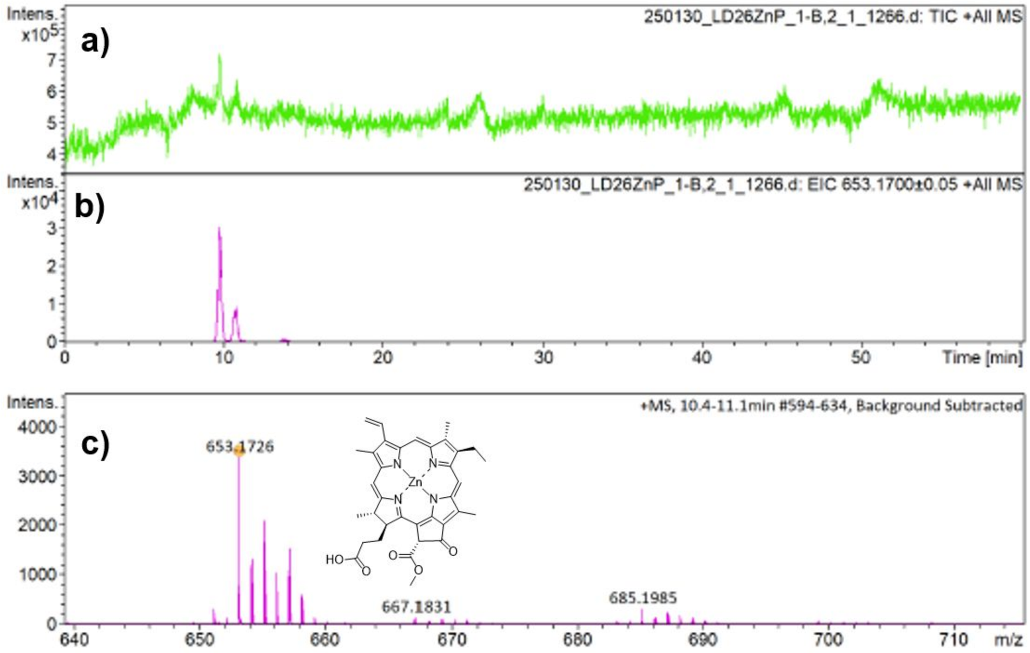

Figure S7. (a) Total Ion Current (TIC) and (b) chromatogram peak obtained by UPLC between 10.4 and 11.1 min with the respective (c) HRMS spectra.

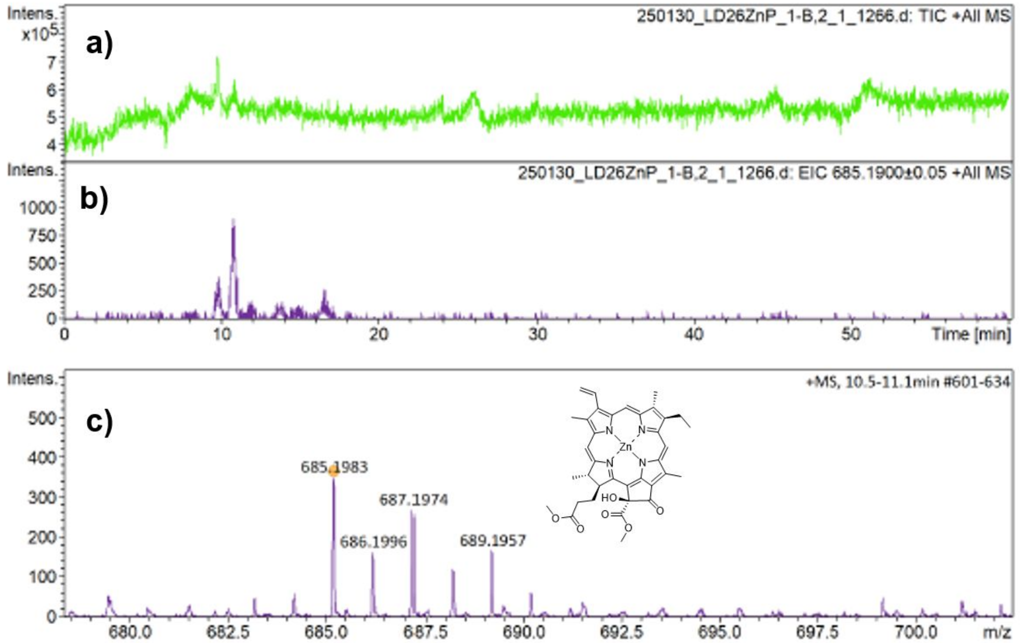

Figure S8. (a) Total Ion Current (TIC) and (b) chromatogram peak obtained by UPLC between 10.5 and 11.1 min with the respective (c) HRMS spectra.

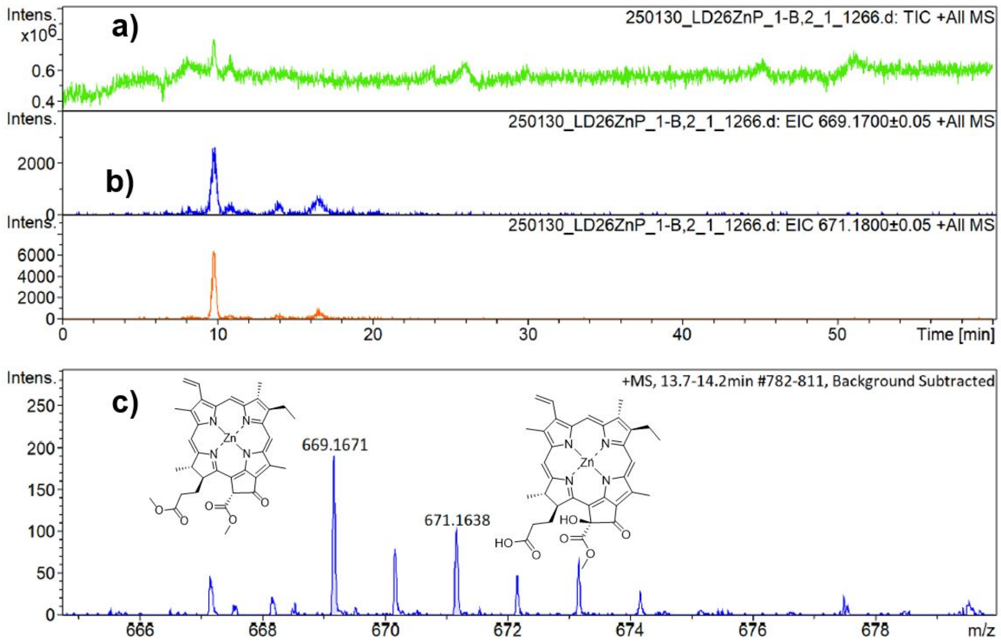

Figure S9. (a) Total Ion Current (TIC) and (b) chromatogram peak obtained by UPLC between 13.7 and 14.2 min with the respective (c) HRMS spectra.

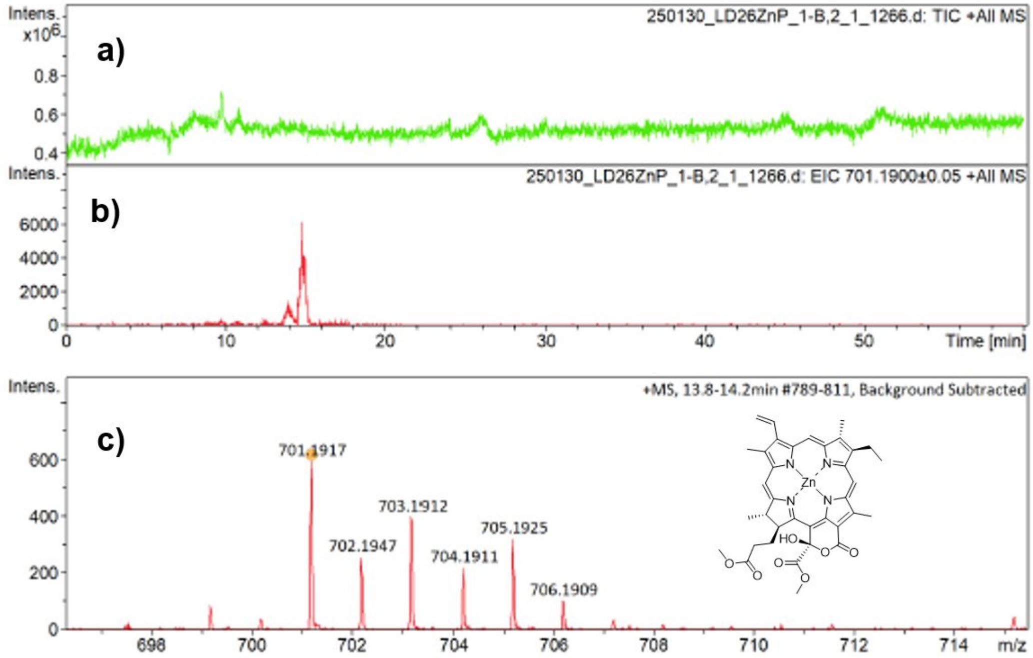

Figure S10. (a) Total Ion Current (TIC) and (b) chromatogram peak obtained by UPLC between 13.8 and 14.2 min with the respective (c) HRMS spectra.

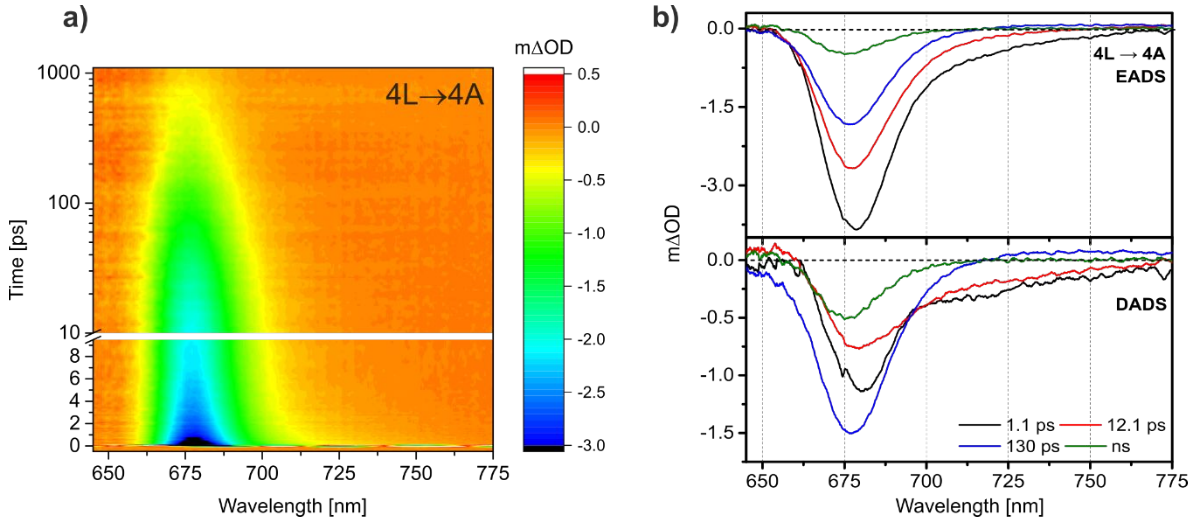

Figure S11. (a) Broadband transient absorption spectroscopy data at magic angle recorded for 4L→4A. (b) Evolution-associated difference spectra (upper panel) and decay-associated difference spectra (bottom panel) retrieved from the global fitting for 4L→4A complex.

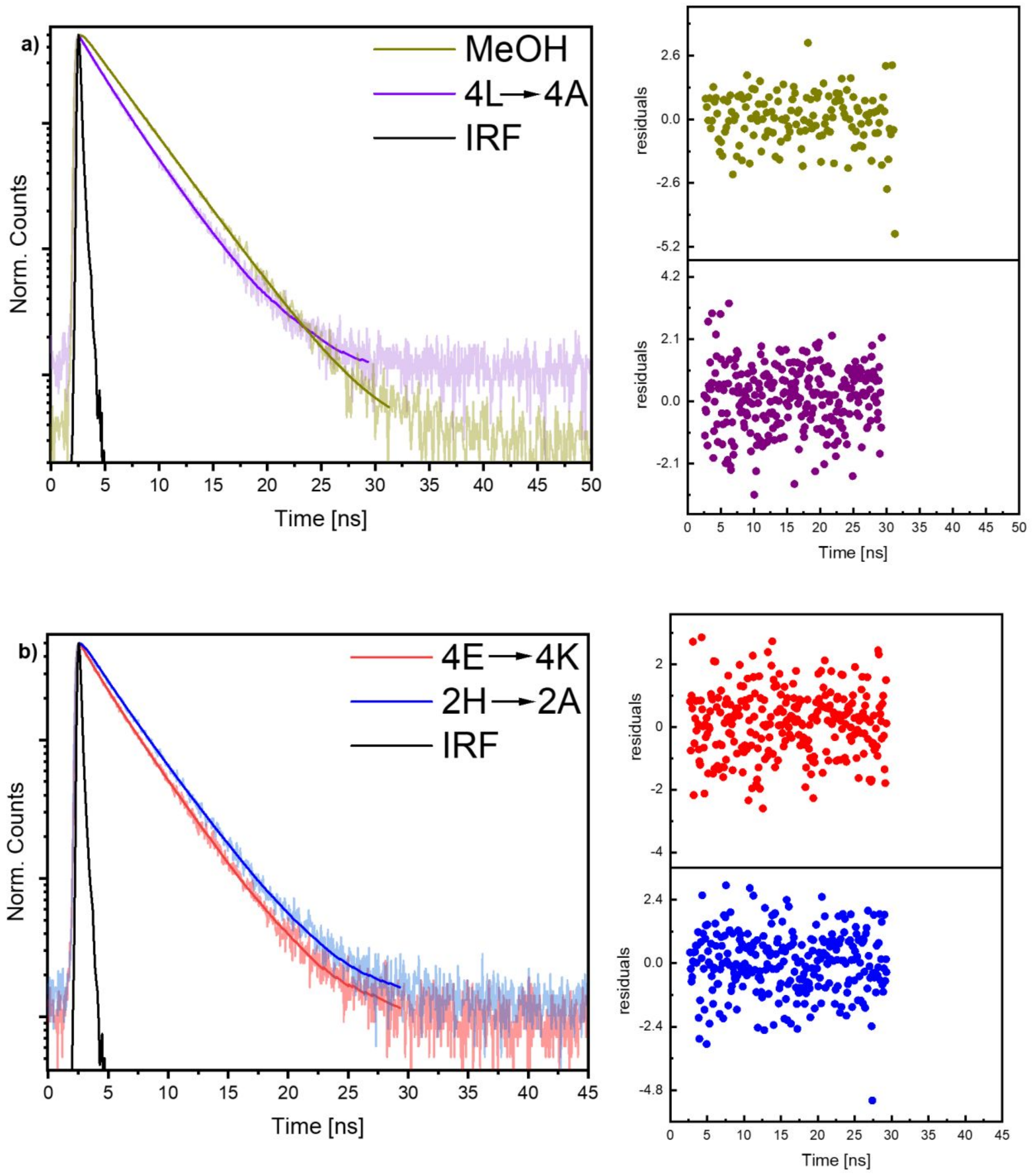

Figure S12. Emission decays of ZnP in (a) methanol, 4L→4A, (b) 2H→2A, 4E→4K complexes.

Table S1 – ZnP emission lifetimes obtained via TCSPC.

Sample	τ [ns]	χ^2
**MeOH**	3.720 ± 0.001	1.186
**2H→2A**	3.685 ± 0.004	1.196
**4E→4K**	3.456 ± 0.003	1.160
**4L→4A**	3.745 ± 0.010	1.166

Supplementary note 2: BB-TAS experiments with parallel polarization

Broadband transient absorption measurements on ZnP in MeOH and the three chromophore-protein assemblies of the study employing parallel polarization of the pump and probe beams are shown in Figure S11. Due to anisotropy effects, parallel polarization data also results in a multiexponential decay for the chromophore in MeOH, but it remains clear that there is a qualitative difference to 2H→2A, in which the DADS of the 2 ps component (τ_1) is significantly blue-shifted with respect to the 139 ps (τ_2) and ns (τ_3) components, while all components coincide in their minimum position for the chromophore in MeOH. Additionally, a faster overall decay hinting at heterogeneity is still visible in the 2H→2A assembly, something that we do not expect to be attributable to anisotropy as rotational speeds of the protein in buffer are expected to be slower than a chromophore in MeOH only.

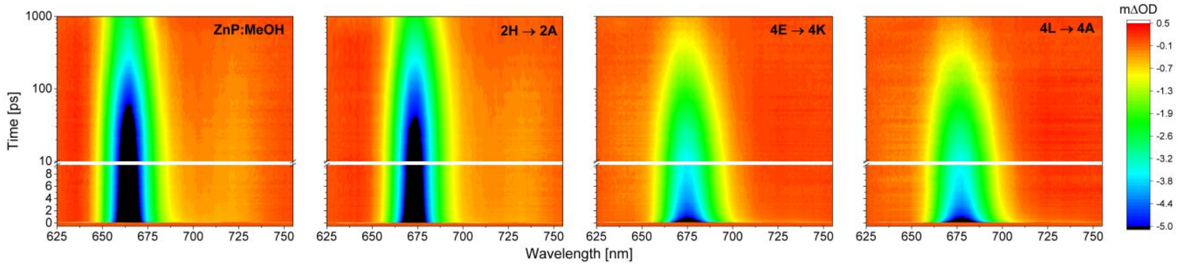

Figure S13. Broadband transient absorption spectroscopy data at parallel relative pump-probe polarization for ZnP in methanol, and 2H→2A, 4E→4K and 4L→4A assemblies.

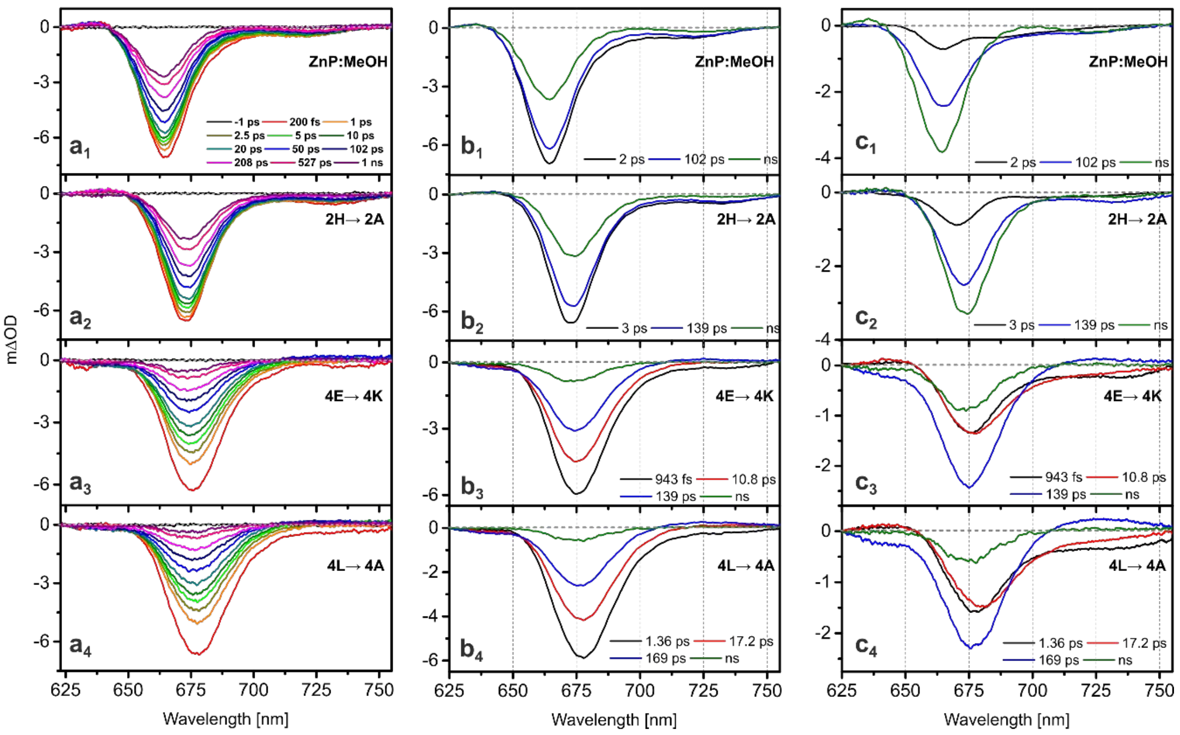

Figure S14. BB-TA spectra collected at characteristic time-delays (a_1-a_4), evolution-associated difference spectra (b_1-b_4) and decay-associated spectra (c_1-c_4) of ZnP in methanol, 2H→2A, 4E→4K and 4L→4A, respectively.

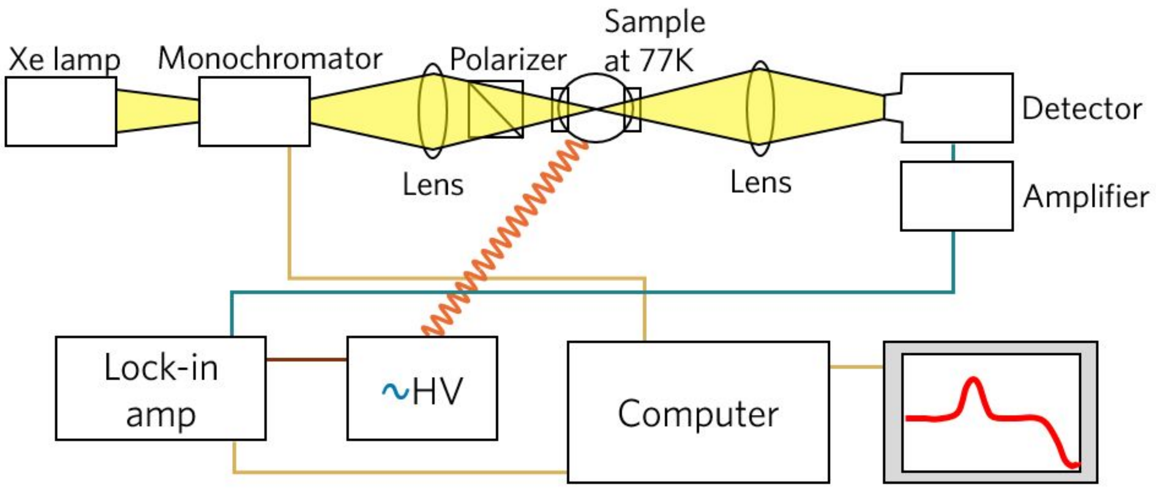

Figure S15. Scheme of the Stark spectroscopy setup employed in this study.

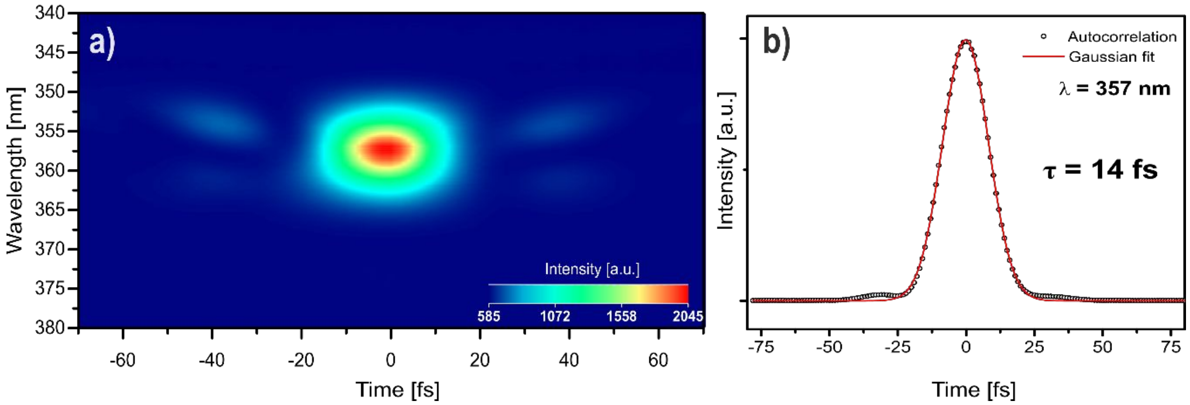

Figure S16. (a) Second-harmonic generation frequency-resolved optical gating (SHG-FROG) profile obtained after NOPA pulse compression, camera baseline is around 580 counts and was not corrected for in contour. (b) Horizontal trace showing the baseline-corrected autocorrelation signal (integral of contour in (a) along frequency dimension, black circles) with a Gaussian fit (red line).

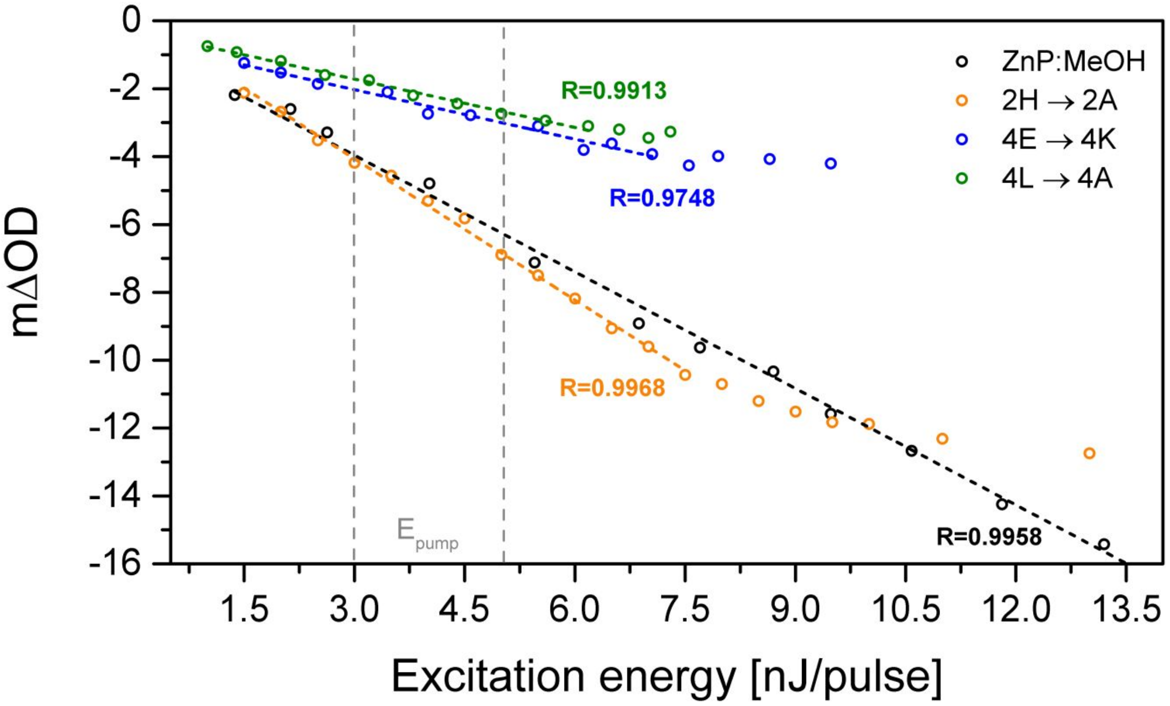

Figure S17. Dependency of the TA signal collected at 675 nm (665 nm for ZnP in methanol) probe wavelength and 100 ps time-delay with respect to excitation pulse energy for the different studied compounds.

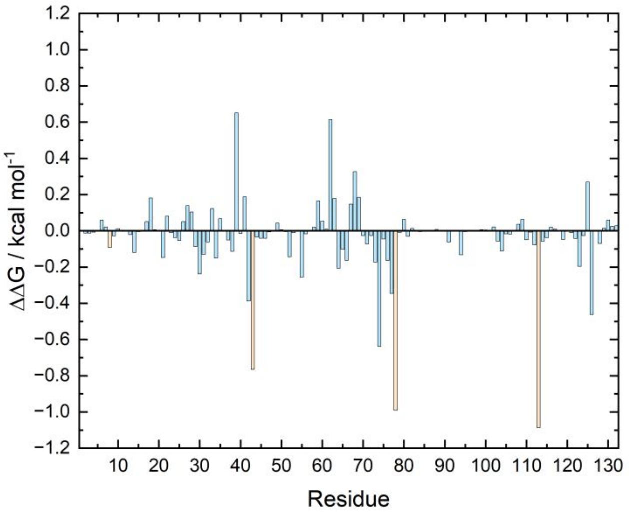

Figure S18. Decomposition of the change in binding energy per residue, when changing from BT6 to the 4E→4K mutant. The residues highlighted in orange are the mutated ones. Negative values indicate that the contribution to binding from a residue is more favorable in the mutant.

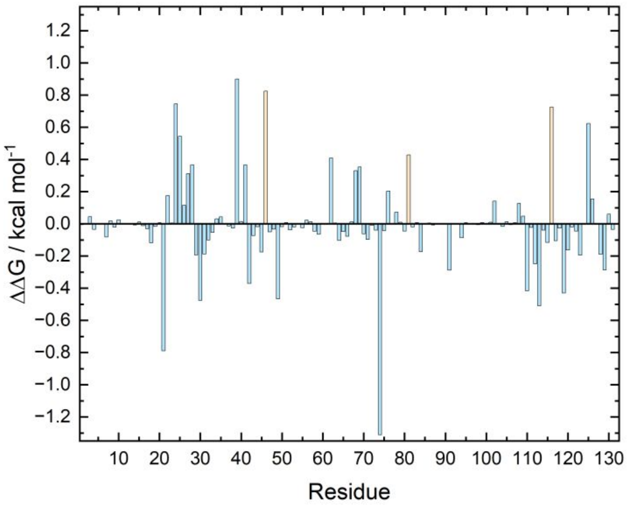

Figure S19. Decomposition of the change in binding energy per residue, when changing from BT6 to the 4L→4A mutant. The residues highlighted in orange are the mutated ones. Negative values indicate that the contribution to binding from a residue is more favorable in the mutant.

Table S2. Decomposition of the QM/MM-PBSA binding energy on its energy components, obtained from MD simulations.

	BT6-4E→4K *vs.* BT6	BT6-4L→4A *vs.* BT6
**Van der Waals term / kcal mol^{-1}**	-3.1	-0.3
**Electrostatic interactions + Polar solvation terms / kcal mol^{-1}**	+6.1	-1.9
**Nonpolar solvation energy term / kcal mol^{-1}**	-0.7	+0.1
**QM/MM interaction energy term / kcal mol^{-1}**	-4.3	-0.4

Table S3. Structural parameters for the three chromophore – protein complexes under study, obtained from MD simulations. In parenthesis, the difference in each parameter for the mutants with respect to BT6.

	BT6	BT6-4E→4K	BT6-4L→4A
**Radius of gyration / Å**	16.86	16.68 (-0.18)	16.66 (-0.20)
**Surface area / Å^2**	8497	8339 (-158)	8485 (-12)
**Average number of H-bonds between chromophores and protein**	1.45	1.57 (+0.12)	1.64 (+0.19)
